# Mediation effects of DNA methylation and hydroxymethylation on birth outcomes after prenatal per- and polyfluoroalkyl substances (PFAS) exposure in the Michigan mother–infant Pairs cohort

**DOI:** 10.1186/s13148-023-01461-5

**Published:** 2023-03-24

**Authors:** Rebekah L. Petroff, Raymond G. Cavalcante, Elizabeth S. Langen, Dana C. Dolinoy, Vasantha Padmanabhan, Jaclyn M. Goodrich

**Affiliations:** 1 Department of Environmental Health Sciences, School of Public Health, University of Michigan, 1415 Washington Heights, Ann Arbor, MI 48109 USA; 2 Epigenomics Core, Biomedical Research Core Facilities, University of Michigan Medical School, Ann Arbor, MI USA; 3 Department of Obstetrics and Gynecology, University of Michigan Medical School, Ann Arbor, MI USA; 4 Department of Nutritional Sciences, School of Public Health, University of Michigan, Ann Arbor, MI USA; 5 Department of Pediatrics Medical School, University of Michigan, Ann Arbor, MI USA

**Keywords:** DNA methylation, Hydroxymethylation, Epigenomics, Developmental origins of health and disease, Prenatal exposures, Per- and polyfluoroalkyl substances, Children’s health, Preterm birth

## Abstract

**Background:**

Per- and polyfluoroalkyl substances (PFAS) are chemicals that are resistant to degradation and ubiquitous in our environments. PFAS may impact the developing epigenome, but current human evidence is limited to assessments of total DNA methylation. We assessed associations between first trimester PFAS exposures with newborn DNA methylation, including 5-methylcytosine (5-mC) and 5-hydroxymethylcytosine (5-hmC). DNA methylation mediation of associations between PFAS and birth outcomes were explored in the Michigan Mother Infant Pairs cohort. Nine PFAS were measured in maternal first trimester blood. Seven were highly detected and included for analysis: PFHxS, PFOA, PFOS, PFNA, PFDA, PFUnDA, and MeFOSAA. Bisulfite-converted cord blood DNA (*n* = 141) and oxidative-bisulfite-converted cord blood (*n* = 70) were assayed on Illumina MethylationEPIC BeadChips to measure total DNA methylation (5-mC + 5-hmC) and 5-mC/5-hmC. Correcting for multiple comparisons, beta regressions were used to assess associations between levels of PFAS and total methylation, 5-mC, or 5-hmC. Nonlinear mediation analyses were used to assess the epigenetic meditation effect between PFAS and birth outcomes.

**Results:**

PFAS was significantly associated with total methylation (*q* < 0.05: PFHxS—12 sites; PFOS—19 sites; PFOA—2 sites; PFNA—3 sites; PFDA—4 sites). In 72 female infants and 69 male infants, there were sex-specific associations between five PFAS and DNA methylation. 5-mC and 5-hmC were each significantly associated with thousands of sites for PFHxS, PFOS, PFNA, PFDA, PFUnDA, and MeFOSAA (*q* < 0.05). Clusters of 5-mC and 5-hmC sites were significant mediators between PFNA and PFUnDA and decreased gestational age (*q* < 0.05).

**Conclusions:**

This study demonstrates the mediation role of specific types of DNA methylation on the relationship between PFAS exposure and birth outcomes*.* These results suggest that 5-mC and 5-hmC may be more sensitive to the developmental impacts of PFAS than total DNA methylation.

**Supplementary Information:**

The online version contains supplementary material available at 10.1186/s13148-023-01461-5.

## Introduction

Gestational exposure to toxicants can negatively impact birth outcomes and have lasting effects on child and adult health, including adverse effects on neurodevelopment, growth, adiposity, and metabolism [1, 2]. One group of toxicants concerning to the health of pregnant women and children are per- and polyfluoroalkyl substances (PFAS), a class of over 12,000 unique chemicals [3] that are widely found in products including cookware, carpet, and food packaging because of their resistance to stains, water, and grease [4, 5]. PFAS have also been used in aqueous film-forming foams used for fire suppression at airports and military bases, leading to the contamination of the surrounding environment and nearby drinking water [6, 7].

PFAS are highly persistent and have accordingly been detected in maternal or umbilical cord plasma or serum in birth cohorts across the United States of America [8–11], Spain [12], China [13], Taiwan [14], Japan [15], and more. Most research to date has measured legacy PFAS, including perfluorohexanesulphonic acid (PFHxS), perfluorooctanesulfonic acid (PFOS), perfluorooctanoic acid (PFOA), and perfluorononanoic acid (PFNA), reporting near ubiquitous detection of all four chemicals in pregnant participants. In other studies, these exposures have been connected with a variety of adverse birth outcomes, including preterm birth or shorter gestational length [16–18], lower birth weight [11, 17–19], and hypertensive disorders of pregnancy, such as preeclampsia [20]. Concerningly, the health effects of prenatal PFAS exposures appear to extend beyond birth, with longitudinal studies reporting links between gestational PFAS and childhood adiposity/metabolic health. In Project Viva (*n* = 876), girls who had higher prenatal exposure to PFHxS, PFOS, PFOA, and PFNA also had increased mid-childhood adiposity [10]. In the HOME study (*n* = 212), early gestational PFHxS and PFOA concentrations were associated with higher central adiposity and increased risk for overweight/obesity at 12 years of age [21].

One major mechanism by which PFAS may be causing birth and later childhood health effects is via epigenetic perturbations. Epigenetic marks are mitotically heritable modifications to DNA and chromatin that control the expression of genes without altering the DNA sequence [22]. During embryogenesis, the epigenome is highly vulnerable to dysregulation, due to post-fertilization epigenetic erasure and post-implantation reprogramming [22]. Any epigenetic disruption during this early developmental stage can be passed on to all subsequent cells across tissue types. One mechanism of epigenetic regulation that is stable across time is DNA methylation at cytosine-guanine (CpG) dinucleotides [23].

DNA methylation at so-called CpG sites (5-methylcytosine or 5-mC) can undergo oxidation to hydroxymethylation (5-hydroxymethylcytosine or 5-hmC) [24, 25] via TET enzymes [26, 27]. While 5-hmC is less abundant than 5-mC, it is a stable DNA modification, with detectable levels in the mammalian brain, liver, kidney, testes, placenta, colon, blood, and embryonic stem cells [28–32]. Like 5-mC, 5-hmC undergoes dynamic changes during early gestation that may persist throughout the lifespan, but the functional roles of 5-hmC and 5-mC in gene regulation seem to be distinct [33]. Still, both types of methylation are independently essential in processes of cell differentiation, fetal growth, and nervous system development and function from early life through adolescence [34–38].

PFAS exposures in human, animal (rodents and zebrafish), and in vitro models have broadly been linked with differences in DNA methylation (for reviews, see Kim et al. [39] and Perng et al*.* [40]). Specifically, human prenatal exposures to PFHxS, PFOS, PFOA, PFNA, and perfluorodecanoic acid (PFDA) have been associated with differences in newborn or childhood blood methylation in six different epidemiological studies assessed via Infinium arrays [40–46]. Across these cohorts, only one statistically significant gene, *TNXB,* was replicated in two studies [45, 46], but differential methylation of genes and enriched gene pathways related to developmental processes, adiposity, metabolism, and neurological function were identified in most studies. There is also evidence for sex-specific associations in two of three studies that considered these relationships [44–46]. However, these epidemiological studies only measured total DNA methylation; the commonly used bisulfite-treatment methods do not distinguish between 5-mC and 5-hmC. In vitro studies have shown that PFAS can disrupt the regulation of oxidating TET genes [27, 47], suggesting that PFAS could broadly alter 5-hmC. Thus, it is important to assess the hydroxymethylome in studies of gestational PFAS exposure.

The present study aimed to identify genes in umbilical cord blood DNA that are differentially methylated and/or hydroxymethylated by first trimester exposures to PFAS and assess how these epigenetic differences mediate relationships between PFAS and adverse birth outcomes in the Michigan Mother–Infant Cohort (MMIP). We hypothesized that gestational exposures to well-studied, legacy PFAS (PFHxS, PFOS, PFOA, and PFNA), as well as additional, less-studied PFAS (PFDA, perfluoroundecanoic acid (PFUnDA), 2-(N-methyl-perfluorooctane sulfonamido) acetic acid (MeFOSAA)) would be associated with altered newborn DNA total methylation, 5-mC, and 5-hmC. We also hypothesized that some associations would be specific to assigned sex at birth.

## Results

### Cohort characteristics

**Fig. 1:**
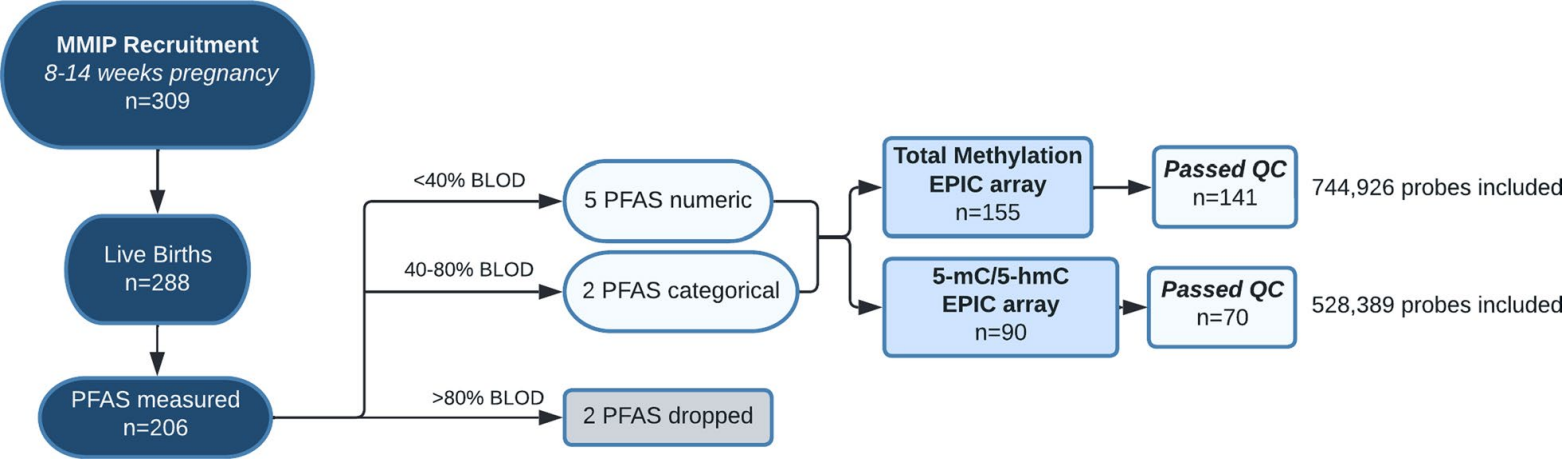
Schematic diagram of study. 309 pregnant people were recruited in the first trimester and 288 remained in the study and had data collected at the time of birth. Among these, 173 provided a cord blood sample for epigenetic analysis at delivery. 206 of these families also had PFAS measured on their first-trimester plasma samples. Analytes of 9 PFAS were measured (Additional file 1: Table S1). Two PFAS were dropped from analysis due to poor detection (> 80% of samples below the limit of detection, BLOD). Another two PFAS (PFUnDA and MeFOSAA) were converted into categorical variables, detected or not detected, due to their moderate detection (> 40% and < 80% of samples BLOD). Five PFAS (PFHxS, PFOS, PFOA, PFNA, PFDA) were treated as continuous concentration measures in analysis, with < 40% of samples BLOD. From dyads that had PFAS, 155 had EPIC data on total methylation, and 90 had 5-hydroxymethylcytosine (5-hmC)/5-methylcytosine (5-mC) EPIC data. For total methylation, 141 samples and 744,926 probes passed quality control (QC). For 5-hmC/5-mC, 70 samples and 528,389 probes passed QC and screening criteria. Abbreviations: 5-hmC: 5-hydroxymethylcytosine; 5-mC: 5-methylcytosine; BLOD: below the limit of detection; MeFOSAA: 2-(N-methyl-perfluorooctane sulfonamido) acetic acid; MMIP: Michigan Mother Infant Pairs; PFAS: per-/polyfluoroalkyl substances; PFHxS: perfluorohexanesulphonic acid; PFDA: perfluorodecanoic acid; PFNA: perfluorononanoic acid; PFOA: perfluorooctanoic acid; PFOS: perfluorooctanesulfonic acid; PFUnDA: perfluoroundecanoic acid; QC: quality control

After samples from the cohort were assessed for quality (Fig. 1), demographic data suggested that maternal variables were largely similar between the entire cohort, those with passing total methylation data, and those with individual level 5-mC and 5-hmC data (Table 1). In those with passing total methylation and those with individual 5-mC/5-hmC data, maternal age was an average of 31.8 years, mean baseline weight was between 69 and 70 kg, and average baseline BMI ranged from 25.5 to 25.8. Participants were largely married, never-smokers, and self-identified as White, non-Hispanic. There were slight differences between 5-mC/5-hmC and both the entire cohort and those with passing total methylation in marriage status, smoking status, and self-reported race and ethnicity (*p* < 0.05).

**Table 1:** Cohort demographics

Maternal characteristics	Mean (SD) or percent (count)		
	Entire cohort *n* = 309*	Total methylation *n* = 141	5-mC + 5-hmC *n* = 70
Age (years)	31.53 (4.2)	31.84 (4.1)	31.80 (4.4)
Parity (count)	1.05 (1.0)	1.01 (0.9)	0.90 (0.8)
Pre-pregnancy weight (kg)	69.28 (15.2)	70.65 (16.9)	69.10 (15.7)
Pre-pregnancy BMI	25.42 (5.3)	25.80 (5.7)	25.51 (5.4)
*Marital status*			
Married	81.6% (252)	80.1% (113)	75.7% (53)
Single	16.8% (52)	19.1% (27)	22.9% (16)
Unknown	1.6% (5)	< 1% (1)	< 1% (1)
*Smoking status*			
Never	69.6% (215)	75.1% (106)	68.6% (48)
Quit before Pregnancy	12.6% (39)	13.4% (19)	12.9% (9)
Quit during Pregnancy	2.3% (7)	2.8% (4)	4.3% (3)
Smoked < *11 cigarettes/day*	2.9% (9)	2.1% (3)	4.3% (3)
Unknown	12.6% (39)	6.4% (9)	10.0% (7)
*Ethnicity and race*			
White, Non-Hispanic	81.9% (253)	81.6% (115)	72.9% (51)
Black or African American, Non-Hispanic	6.5% (20)	6.4% (9)	10.0% (7)
Hispanic	2.6% (8)	2.1% (3)	4.3% (3)
Asian, Non-Hispanic	4.5% (14)	2.8% (4)	2.9% (2)
Native American, Non-Hispanic	0.6% (2)	< 1% (1)	1.4% (1)
Hawaiian or Pacific Islander	0.6% (2)	0% (0)	0% (0)
Multi-Ethnic/Racial	1.0% (3)	2.1% (3)	1.4% (1)
Unknown	2.3% (7)	4.3% (6)	5.7% (4)

Infant Characteristics	*n* = 288		
Sex			
Male	48.3% (147)	48.9% (69)	52.9% (37)
Female	51.0% (139)	51.1% (72)	47.1% (33)
Gestational Age at Birth (days)	274.49 (12.5)	276.73 (8.1)	275.86 (9.1)
Birthweight (grams)	3414.3 (533.0)	3435.0 (482.1)	3351.79 (518.9)
Fenton Z-Score	0.09 (0.91)	0.04 (0.91)	−0.11 (1.0)

PFAS Exposures^	*n* = 206		
PFHxS (µg/L)	3.40 (2.0)	3.19 (1.6)	3.08 (1.7)
PFOS (µg/L)	5.73 (2.9)	5.25 (1.7)	5.04 (1.8)
PFOA (µg/L)	1.35 (0.9)	1.14 (1.9)	1.14 (1.9)
PFNA (µg/L)	0.41 (0.2)	0.36 (1.8)	0.37 (1.8)
PFDA (µg/L)	0.16 (0.1)	0.13 (1.8)	0.12 (1.8)
PFUnDA (% above the LOD)	41.3% (85)	35.5% (50)	38.6% (27)
MeFOSAA (% above the LOD)	34% (70)	37.6% (53)	42.9% (30)

5-hmC: 5-hydroxymethylcytosine; 5-mC: 5-methylcytosine; BMI: body mass index; LOD: limit of detection; MeFOSAA: 2-(N-methyl-perfluorooctane sulfonamido) acetic acid; PFAS: per-/polyfluoroalkyl substances; PFHxS: perfluorohexanesulphonic acid; PFDA: perfluorodecanoic acid; PFNA: perfluorononanoic acid; PFOA: perfluorooctanoic acid; PFOS: perfluorooctanesulfonic acid; PFUnDA: perfluoroundecanoic acid.

*This includes all women originally enrolled in the first trimester. Note that many were lost to follow-up or provided incomplete data. Of these, 288 stayed in the study and had a live, singleton birth at the study hospital (2 were missing information on infant sex). A subset of 206 participants with archived first trimester samples were selected for the PFAS analysis.

^Numeric PFAS reported in geometric means.

### PFAS exposure assessment

PFHxS, PFOS, PFOA, and PFNA were highly detected, with > 89% of measurements above the LODs (Additional file 1: Table S1). PFDA was well detected, with 60% of measurements above the LOD (Additional file 1: Table S1). All five of these PFAS were treated as numeric variables in analysis. Geometric mean concentrations were 3.2 µg/L (GSD: 1.6) for PFHxS; 5.3 µg/L (GSD: 1.7) for PFOS; 1.1 µg/L (GSD: 1.9) for PFOA; 0.37 µg/L (GSD: 1.8) for PFNA; and 0.12 µg/L (GSD: 1.8) for PFDA (Additional file 1: Table S1, Fig. S2). PFUnDA and MeFOSAA were moderately detected, with 35.5% and 37.6% of samples above the LOD (Additional file 1: Table S1). These were treated as categorical variables in the final analysis.

Intercorrelation of PFAS (Additional file 1: Fig. S3) showed that there were significant correlations between PFHxS and PFOS (*r*^2^: 0.29, *p* < 0.001) and PFOA (*r*^2^: 0.49, *p* < 0.001); PFOS and PFDA (*r*^2^: 0.52, *p* < 0.001); and PFNA and PFOS (*r*^2^: 0.77, *p* < 0.001), PFOA (*r*^2^: 0.58, *p* < 0.001), and PFDA (*r*^2^: 0.73, *p* < 0.001). Using Chi-squared tests, PFUnDA and MeFOSAA were not related to other PFAS (*p* > 0.05).

Parity was negatively correlated with concentrations of PFOS (*r*^2^: −0.40, *p* < 0.001) and PFOA (*r*^2^: −0.29, *p* < 0.001). Maternal age and pre-pregnancy BMI were not correlated with any PFAS (*p* > 0.05). Race and ethnicity, smoking status, and marital status each had associations with 1–2 PFAS: self-reported race as African American or Black (but not other self-reported race or ethnicities or missing self-reported race or ethnicity) was associated with PFUnDA below the LOD (*χ*^2^: 64, *p* < 0.05); smoking was associated with MeFOSAA below the LOD(*χ*^2^: 169, *p* < 0.05); and singleness was associated with MeFOSAA below the LOD (*χ*^2^: 188, *p* < 0.001). Due to these results, self-reported race was included in models as a variable for reporting African American or Black as a proxy for specific racism and racist policies that influence PFAS exposure burden in this self-identified group.

### PFAS and total DNA methylation

**Fig. 2:**
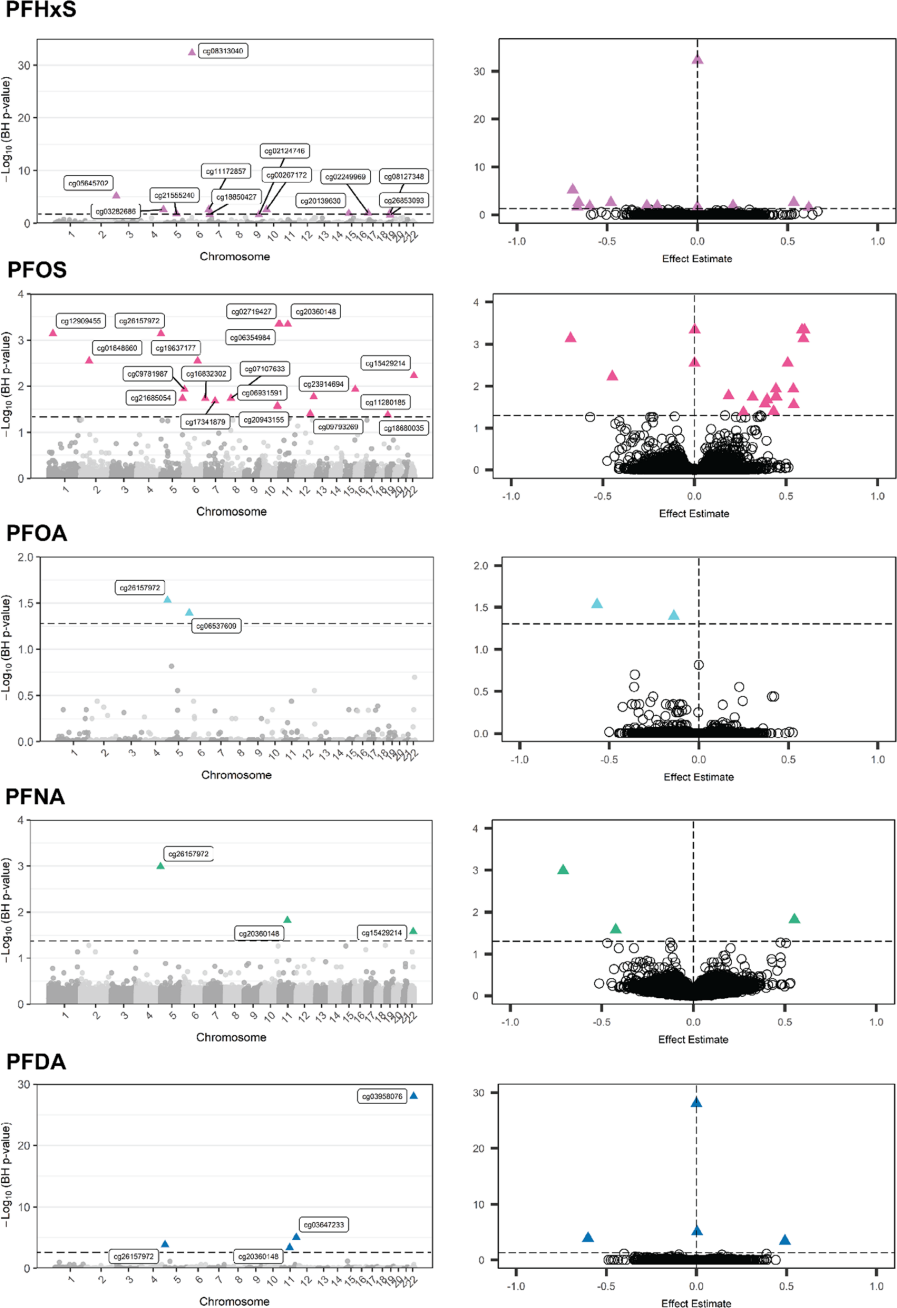
Significant total methylation sites (n = 141). Each row represents an individual PFAS. On the left, Manhattan plots describe the chromosomal location of all sites and corresponding Benjamini–Hochberg corrected *p*-value (*q*-value), with the sites significantly associated with PFAS labeled and noted with solid triangles. On the right, volcano plots depict all PFAS-site associations by effect estimate representing differences for each log-fold unit change in PFAS concentration or categorical PFAS detection and Benjamini–Hochberg corrected *p*-value (*q*-value), with the statistically significant sites noted in solid triangles. Dashed lines on both represent *q*-value = 0.05. Abbreviations: PFAS: per-/polyfluoroalkyl substances; PFHxS: perfluorohexanesulphonic acid; PFDA: perfluorodecanoic acid; PFNA: perfluorononanoic acid; PFOA: perfluorooctanoic acid; PFOS: perfluorooctanesulfonic acid

In the models for DNA methylation, parity, self-reported race (as Black or African American), and smoking status were considered true confounders. Marital status was not included in the final model, as this variable has not been traditionally associated with effects or differences in infant DNA methylation. Beta regression models for total methylation across 744,926 CpG sites were fit for each PFAS, and genomic inflation factors for each model suggested minimal *p*-value inflation (Additional file 1: Table S3). Site-specific differences in total methylation were found for all PFAS modeled as continuous concentrations, including in 12 sites for PFHxS, 19 sites for PFOS, 2 sites for PFOA, 3 sites for PFNA, and 4 sites for PFDA (*q* < 0.05, Fig. 2, see Tables 2 and 3). Of these, total methylation of several CpG sites overlapped between PFAS—cg15429214 in an intergenic region of chromosome 22 was negatively associated with PFOS and PFNA; cg20360148 on the *ATG2A* (autophagy related 2A) gene was positively associated with PFOS, PFNA, and PFDA; and cg26157972 on an intergenic region of chromosome 5 was negatively associated with PFOA, PFOS, PFNA, and PFDA (Table 3). PFHxS was also positively associated with total methylation at two sites near the transcription start site of *GTPBP3* (GTP binding protein 3, mitochondrial), but no other PFAS had significant sites in this gene.

**Table 2:** Number of Sites Significantly Associated with PFAS by Model (q < 0.05)

	Total Methylation (*n* = 141)	Sex Interaction (*n* = 141)	Females (*n* = 72)	Males (*n* = 69)
PFHxS	12	84	17	81
PFOS	19	98	78	10
PFOA	2	1	0	0
PFNA	3	5	1	0
PFDA	4	4	2	2
PFUnDA	0	2	2	2
MeFOSAA	0	1	0	0

*Counts of the significant number of DNA methylation sites related to each PFAS exposure. Sex interaction models were used to select the sites with evidence for sex-specific relationships. Models were then run at these sites, stratified by infant sex. The following models were fit, where bolded term indicates the estimate of interest generating the counts above:*

$$\begin{aligned} {\text{Total}}\;{\text{Methylation}}\;{\text{at}}\;{\text{744}},{\text{926}}\;{\text{sites}} & = \beta _{0} + \varvec{\beta} _{1} {\mathbf{PFAS}} + \beta _{2} {\text{Parity}} + \beta _{3} {\text{Smoking}} + \beta _{4} {\text{Race}} \\ & \quad + \beta _{5} {\text{CD}}4{\text{T}} + \beta _{6} {\text{CD}}8{\text{T}} + \beta _{7} {\text{GranCell}} \\ & \quad + \beta _{8} n{\text{RBC}} + \beta _{9} {\text{PC}}1 + \beta _{{10}} {\text{PC}}2 + \beta _{{12}} {\text{Sex}} \\ \end{aligned}$$

$$\begin{aligned} {\text{Sex}}\;{\text{Interaction}}\;{\text{Methylation}}\;{\text{at}}\;{\text{744}},{\text{926}}\;{\text{sites}} = & \beta _{0} + \beta _{1} {\text{PFAS}} + \beta _{2} {\text{Parity}} + \beta _{3} {\text{Smoking}} + \beta _{4} {\text{Race}} \\ & \quad + \beta _{5} {\text{CD}}4{\text{T}} + \beta _{6} {\text{CD}}8{\text{T}} + \beta _{7} {\text{GranCell}} + \beta _{8} n{\text{RBC}} \\ & \quad + \beta _{9} {\text{PC}}1 + \beta _{{10}} {\text{PC}}2 + \beta _{{12}} {\text{Sex}} + \varvec{\beta }_{{{\mathbf{13}}}} {\mathbf{Sex*PFAS}} \\ \end{aligned}$$

$$\begin{aligned} {\text{Male}}\;{\text{or}}\;{\text{Female}}\;{\text{Methylation}}\;{\text{at}}\;{\text{Sites}}\;{\text{with}}\;{\text{Sex}}\;{\text{Interaction}} & = \beta _{0} + \varvec{\beta }_{{\mathbf{2}}} {\mathbf{PFAS}} + \beta _{2} {\text{Parity}} + \beta _{3} {\text{Smoking}} + \beta _{4} {\text{Race}} \\ & \quad + \beta _{5} {\text{CD}}4{\text{T}} + \beta _{6} {\text{CD}}8{\text{T}} + \beta _{7} {\text{GranCell}} \\ & \quad + \beta _{8} n{\text{RBC}} + \beta _{9} {\text{PC}}1 + \beta _{{10}} {\text{PC}}2 \\ \end{aligned}$$

CD4T: CD4 T lymphocytes; CD8T: CD8 T lymphocytes; GranCell: granulated cells; MeFOSAA: 2-(N-methyl-perfluorooctane sulfonamido) acetic acid; nRBC: nucleated red blood cells; PC: principal component representing batch effects; PFAS: per-/polyfluoroalkyl substances; PFHxS: perfluorohexanesulphonic acid; PFDA: perfluorodecanoic acid; PFNA: perfluorononanoic acid; PFOA: perfluorooctanoic acid; PFOS: perfluorooctanesulfonic acid; PFUnDA: perfluoroundecanoic acid

**Table 3:** Sites with significant associations between PFAS and total methylation (*q* < 0.05, *n* = 141)

PFAS	Illumina CpG Name	Estimate	SE	Lower 95% CI	Upper 95% CI	*p*-value	BH *q*-value	Chromosome: Position	UCSC Gene Name
*PFHxS*									
	cg05645702	−0.692	0.094	−0.876	−0.508	1.71E−11	6.37E−06	chr2:242190905	*HDLBP*
	cg03282686	0.533	0.088	0.361	0.705	1.56E−08	0.003	chr4:177116826	*SPATA4*
	cg21555240	−0.598	0.109	−0.812	−0.384	1.93E−07	0.016	chr5:89734950	
	cg08313040	< 0.001	< 0.001	< 0.001	< 0.001	6.70E−39	4.99E−33	chr6:33092243	*HLA-DPB2*
	cg11172857	−0.659	0.109	−0.873	−0.445	1.73E−08	0.003	chr6:168956903	*SMOC2*
	cg18850427	< 0.001	< 0.001	< 0.001	< 0.001	2.82E−07	0.021	chr7:4009036	*SDK1*
	cg02124746	−0.666	0.124	−0.909	−0.423	3.54E−07	0.024	chr9:91615137	*S1PR3*
	cg00267172	−0.479	0.079	−0.634	−0.324	1.14E−08	0.003	chr10:11487791	
	cg20139630	−0.222	0.040	−0.300	−0.144	1.48E−07	0.014	chr15:40476003	*BUB1B*
	cg02249969	−0.280	0.050	−0.378	−0.182	1.11E−07	0.012	chr17:1969338	*SMG6*
	cg08127348	0.197	0.035	0.128	0.266	1.04E−07	0.012	chr19:17448311	*GTPBP3*
	cg26853093	0.616	0.116	0.389	0.843	4.67E−07	0.029	chr19:17448469	*GTPBP3*
*PFOS*									
	cg12909455	0.595	0.095	0.409	0.781	4.91E−09	0.001	chr1:32458635	
	cg01848660	0.509	0.086	0.340	0.678	2.45E−08	0.003	chr2:68269960	*C1D*
	cg26157972	−0.676	0.107	−0.886	−0.466	4.49E−09	0.001	chr5:1049232	
	cg21685054	0.449	0.083	0.286	0.612	2.96E−07	0.018	chr5:169810494	*KCNMB1*
	cg09781987	0.539	0.097	0.349	0.729	1.58E−07	0.012	chr6:4828434	*CDYL*
	cg19637177	0.001	< 0.001	< 0.001	0.003	2.64E−08	0.003	chr6:109417087	*C6orf182*
	cg16832302	0.443	0.082	0.282	0.604	3.30E−07	0.018	chr6:169689336	
	cg17341879	0.397	0.074	0.252	0.542	4.22E−07	0.021	chr7:75690308	*MDH2*
	cg07107633	0.318	0.059	0.202	0.434	3.45E−07	0.018	chr8:40960365	
	cg20943155	0.542	0.103	0.340	0.744	6.33E−07	0.028	chr10:118031958	*GFRA1*
	cg06931591	0.385	0.073	0.242	0.528	5.66E−07	0.026	chr10:118980094	
	cg06354984	0.587	0.090	0.411	0.763	1.41E−09	4.60E−04	chr10:128211107	*C10orf90*
	cg02719427	0.002	< 0.001	< 0.001	0.004	1.85E−09	4.60E−04	chr11:2151725	*INS-IGF2; IGF2*
	cg20360148	0.600	0.092	0.420	0.780	1.69E−09	4.60E−04	chr11:64685078	*ATG2A*
	cg09793269	0.432	0.084	0.267	0.597	9.61E−07	0.040	chr12:105348269	
	cg23914694	0.186	0.034	0.119	0.253	2.51E−07	0.017	chr12:132832250	*GALNT9*
	cg11280185	0.444	0.080	0.287	0.601	1.42E−07	0.012	chr16:237270	
	cg18680035	0.267	0.052	0.165	0.369	1.08E−06	0.042	chr19:6818326	*VAV1*
	cg15429214	−0.448	0.078	−0.601	−0.295	6.43E−08	0.006	chr22:43166281	
*PFOA*									
	cg26157972	−0.569	0.097	−0.759	−0.379	3.95E−08	0.029	chr5:1049232	
	cg06537609	−0.140	0.025	−0.189	−0.091	1.09E−07	0.040	chr5:176217086	
*PFNA*									
	cg26157972	−0.711	0.109	−0.925	−0.497	1.39E−09	0.001	chr5:1049232	
	cg20360148	0.552	0.094	0.368	0.736	4.08E−08	0.015	chr11:64685078	*ATG2A*
	cg15429214	−0.424	0.075	−0.571	−0.277	1.06E−07	0.026	chr22:43166281	
*PFDA*									
	cg26157972	−0.602	0.089	−0.776	−0.428	5.30E−10	1.32E−04	chr5:1049232	
	cg20360148	0.491	0.076	0.342	0.640	2.12E−09	3.95E−04	chr11:64685078	*ATG2A*
	cg03647233	0.003	< 0.001	0.001	0.005	2.33E−11	8.68E−06	chr11:117387430	*DSCAML1*
	cg03958076	< 0.001	< 0.001	< 0.001	< 0.001	1.25E−34	9.30E−29	chr22:41304942	*XPNPEP3*

Because beta regressions (logit link functions) were used to model differences in DNA methylation, estimates and SEs for methylation differences representing each log-fold unit change in PFAS concentration or categorical PFAS detection can be approximated by exp(estimate), exp(SE), or exp(CI)

BH: Benjamini–Hochberg; CI: confidence interval; CpG: cytosine-guanine site where methylation was measured; PFAS: per-/polyfluoroalkyl substances; PFHxS: perfluorohexanesulphonic acid; PFDA: perfluorodecanoic acid; PFNA: perfluorononanoic acid; PFOA: perfluorooctanoic acid; PFOS: perfluorooctanesulfonic acid; SE: standard error

When comparing these estimates to previously published associations between prenatal PFAS and neonatal total methylation, there were not strong similarities (Additional file 1: Fig. S4). There was no overlap in significant CpG sites by PFAS identified in the present and former studies at *q* < 0.05 (Additional file 2: Table S1). At a raw *p*-value < 0.05 for the present study, only a few associations replicated in the same direction as previous PFAS epigenome-wide association studies: PFOS with increased methylation in *ANO3* (cg05146852) [42] and PFNA with decreased methylation in *HIF1A* (cg04509825) and *TTL* (cg03065329) and increased methylation in *PTGIS* (cg27059136) and *USP19* (cg01673931) [41].

When examining the sex-specific differences associated with each PFAS, all PFAS had at least one site with a significant sex interaction (*q* < 0.05, Table 2 and Additional file 2: Table S2). After stratifying by female and male infants and running models for these sites, PFHxS, PFOS, PFNA, PFDA, and PFUnDA all had significant sex-specific associations with total methylation in at least one CpG site (*q* < 0.05, Tables 2, Additional file 1: Tables S4, S5). In females, PFHxS had 17 significant sites, PFOS had 78 sites, PFNA had 1 site, PFNA had 2 sites, and PFUnDA had 2 sites (Additional file 1: Table S4). In males, PFHxS had 81 significant sites, PFOS had 10 sites, PFDA had 2 sites, and PFUnDA had 2 sites (Additional file 1: Table S5). Sex interactions in PFHxS and PFOS were primarily driven by males and females, respectively. Within each sex, most of the significant CpG sites were isolated to unique genes, with the exception of three genes with multiple CpG sites in females that were associated with PFOS (*C2orf78*, chromosome 2 open reading frame 78; *SPATS2L*, spermatogenesis associated serine rich 2 Like; *RAP1GAP2*, RAP1 GTPase activating protein 2), four genes with multiple CpG sites in males that were associated with PFHxS (*SPATA4*, spermatogenesis associated 4; *AGPAT1*, 1-acylglycerol-3-phosphate O-acyltransferase 1; *RNF5*, ring finger protein 5; *RNF5P1*, *RNF5*-pseudogene 1), and one gene that was associated with both PFHxS and PFOS in males (*S1PR3*, sphingosine-1-phosphate receptor 3). *AGPAT1*, *RNF5*, and *RNF5P1* are located near each other on chromosome 6 and were highly associated with PFHxS in males, potentially representing a region of interest that is sensitive to total methylation changes in males. Specific region-wide analyses or pathway analyses, however, were unable to be conducted in any total methylation analysis, as there were too few significant sites overall.

### PFAS and 5-mC and 5-hmC

**Fig. 3:**
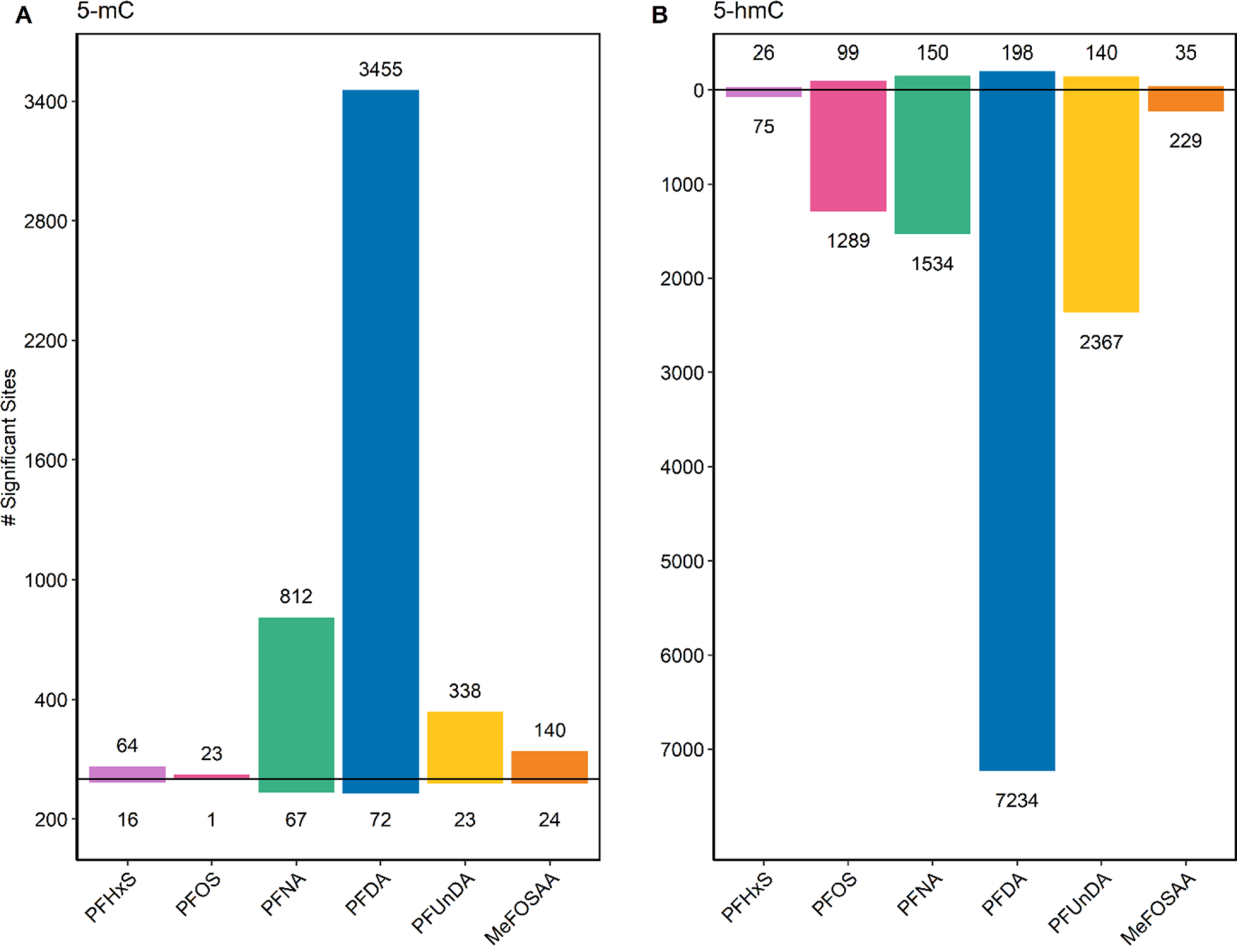
Number and direction of significant associations between PFAS and 5-methylcytosine (5-mC) and 5-hydroxymethylcytosine (5-hmC) after stratification of loci with a significant PFAS: type of methylation interaction (n = 70). **A** shows all sites with a significant association between each PFAS and 5-mC (*q* < 0.05) and **B** shows the 5-hmC sites (*q* < 0.05). Counts above the horizontal line at zero represent the number of sites that were positively associated with each PFAS, and counts below the horizontal line represent the number of sites that were negatively associated with each PFAS. Abbreviations: MeFOSAA: 2-(N-methyl-perfluorooctane sulfonamido) acetic acid; PFAS: per-/polyfluoroalkyl substances; PFHxS: perfluorohexanesulphonic acid; PFDA: perfluorodecanoic acid; PFNA: perfluorononanoic acid; PFOS: perfluorooctanesulfonic acid; PFUnDA: perfluoroundecanoic acid

Using the model outlined above for the interaction of PFAS with type of methylation (5-mC and 5-hmC), beta regression models across 528,389 CpG sites in the genome were fit for each PFAS (*n* = 70), and genomic inflation factors for each model suggested minimal inflation (Additional file 1: Table S6). After filtering sites that had an interaction by methylation type, over 15,000 sites were identified for stratification (*q* < 0.2), including 105 for PFHxS; 1,516 for PFOS; 637 for PFOA; 2,281 for PFNA; 8,054 for PFDA; 3,103 for PFUnDA; and 272 for MeFOSAA (Table 4, Additional file 2: Table S3). Each of these sites was stratified for methylation type, and 5,036 and 13,376 of these sites had significant associations between a PFAS with 5-mC or 5-hmC, respectively (*q* < 0.05, see Table 4). Within each PFAS, there were more sites associated with differences in 5-hmC as compared to 5-mC (Additional file 2: Tables S4 and S5). The majority of significant sites had decreased 5-hmC (75 sites for PFHxS, 1,289 for PFOS; 1,534 for PFNA; 7,234 for PFDA; 2,367 for PFUnDA; and 229 for MeFOSAA) and increased 5-mC (64 for PFHxS; 23 for PFOS; 812 for PFNA; 3,455 for PFDA; 338 for PFUnDA; and 140 for MeFOSAA, see Fig. 3). For all PFAS but PFOA, there were more significant associations with 5-hmC, when compared to 5-mC (Table 4).

**Table 4:** Number of significant associations between PFAS with 5-mC and/or 5-hmC (n = 70)

	5-mC/5-hmC Interaction *(q* < *0.2)*	5-mC Only *(q* < *0.05)*	5-hmC Only *(q* < *0.05)*
PFHxS	105	80	101
PFOS	1516	24	1388
PFOA	637	1	0
PFNA	2281	879	1684
PFDA	8054	3527	7432
PFUnDA	3103	361	2507
MeFOSAA	272	164	264

*Counts of the number of CpG sites with statistically significant associations between each PFAS and methylation (n* = *70 for all analyses). 5-mC/5-hmC-interaction models were used to select the sites to stratify by each type of methylation. The following models were fit, where bolded term indicates the estimate of interest generating the counts above:*

$$\begin{aligned} 5 - {\text{mC}}\;{\text{or}}\;5 - {\text{hmC}}\;{\text{proportion}}\;{\text{at}}\;{\text{528}},{\text{389}}\;{\text{Sites}} & = \beta _{0} + \beta _{1} {\text{PFAS}} + \beta _{2} {\text{Parity}} + \beta _{3} {\text{Smoking}} + \beta _{4} {\text{Race}} \\ & \quad + \beta _{5} {\text{sex}} + \beta _{6} {\text{CD}}4{\text{T}} + \beta _{7} {\text{CD}}8{\text{T}} + \beta _{8} {\text{GranCell}} \\ & \quad + \beta _{9} n{\text{RBC}} + \beta _{{10}} {\text{PC}}1 + \beta _{{11}} {\text{PC}}2 + \beta _{{12}} {\text{Type}} \\ & \quad + \varvec{\beta }_{{{\mathbf{13}}}} {\mathbf{Type*PFAS}} + [1|ID] \\ \end{aligned}$$

$$5 - {\text{mC}}\;{\text{or}}\;5 - hmC\;{\text{Methylation}}\;{\text{at}}\;{\text{Sites}}\;{\text{with}}\;{\text{Type}} - {\text{Interaction}} \, = \, \beta _{0} + \varvec{\beta }_{{\mathbf{1}}} {\mathbf{PFAS}} + \beta _{2} {\text{Parity}} + \beta _{3} {\text{Smoking}} + \beta _{4} {\text{Race}} + \beta _{5} {\text{CD4T}} + \beta _{6} {\text{CD}}8{\text{T}} + \beta _{7} {\text{GranCell}} + \beta _{8} n{\text{RBC}} + \beta _{9} {\text{PC}}1 + \beta _{{10}} {\text{PC}}$$

CD4T: CD4 T lymphocytes; CD8T: CD8 T lymphocytes; GranCell: granulated cells; MeFOSAA: 2-(N-methyl-perfluorooctane sulfonamido) acetic acid; nRBC: nucleated red blood cells; PC: principal component representing batch effects; PFAS: per-/polyfluoroalkyl substances; PFHxS: perfluorohexanesulphonic acid; PFDA: perfluorodecanoic acid; PFNA: perfluorononanoic acid; PFOA: perfluorooctanoic acid; PFOS: perfluorooctanesulfonic acid; PFUnDA: perfluoroundecanoic acid

**Fig. 4:**
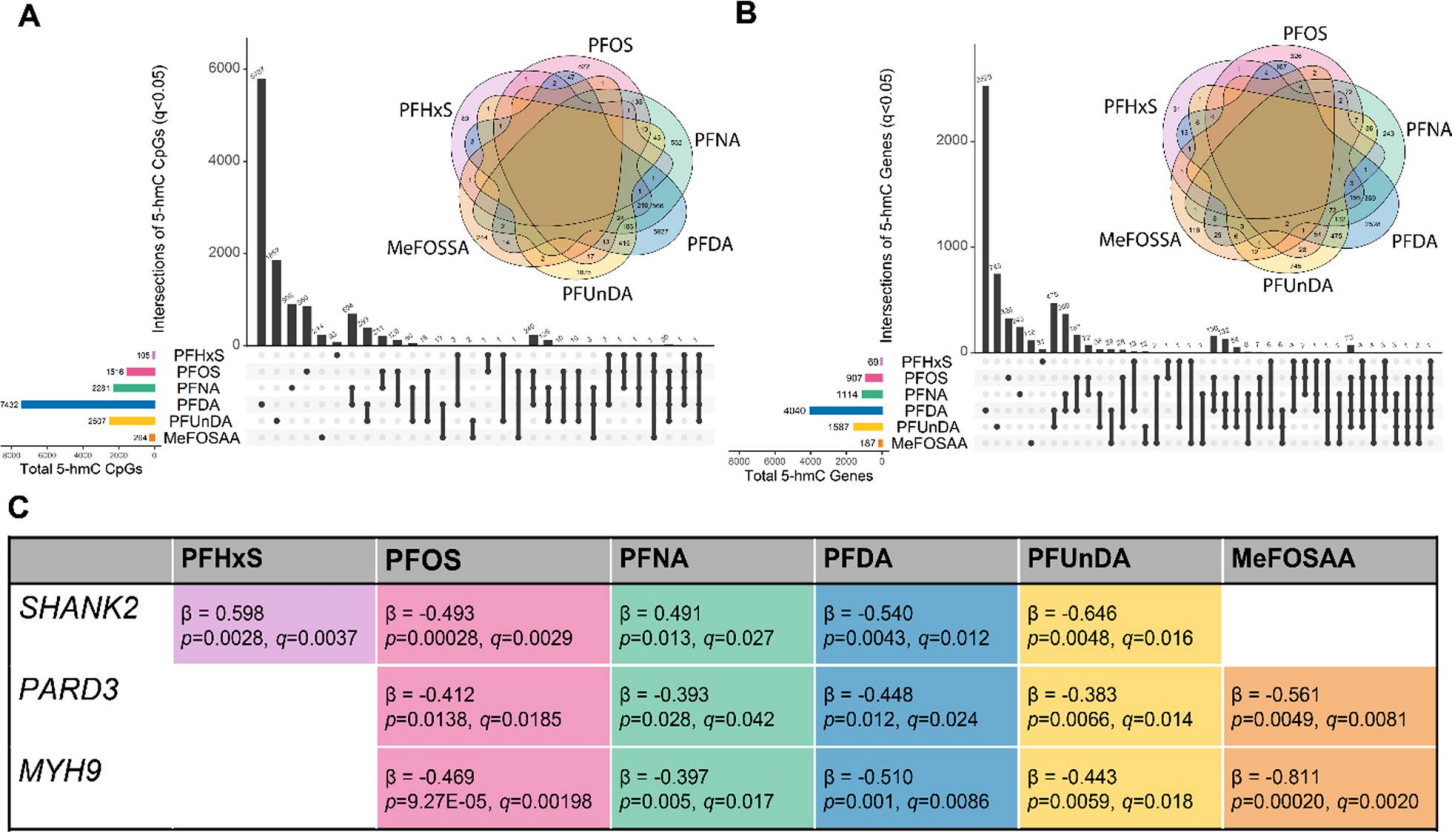
Overlap for significant 5-hydroxymethylcytosine (5-hmC, *q* < 0.05) sites (**A**) and genes (**B**) by PFAS (*n* = 70). Each plot and Venn diagram inset shows the overlap between all PFAS in the present study. **C** Lists the three genes that overlapped between at least five PFAS. β corresponds the beta regression coefficient estimate representing exp(estimate) differences for each log-fold unit change in PFAS concentration or categorical PFAS detection, p represents the uncorrected *p*-value, and *q* represents the Benjamini–Hochberg corrected *q*-value. Abbreviations: MeFOSAA: 2-(N-methyl-perfluorooctane sulfonamido) acetic acid; *MYH9*: myosin heavy chain 9; *PARD3:* Par-3 family cell polarity regulator; PFAS: per-/polyfluoroalkyl substances; PFHxS: perfluorohexanesulphonic acid; PFDA: perfluorodecanoic acid; PFNA: perfluorononanoic acid; PFOS: perfluorooctanesulfonic acid; PFUnDA: perfluoroundecanoic acid; *SHANK2*: SH3 and multiple ankyrin repeat domains 2

Accordingly, there was little overlap in CpG sites or genes between PFAS for 5-mC; only the gene *RPS6KA2* (ribosomal protein S6 kinase A2) had overlap between more than 3 PFAS (PFNA, PFDA, PFUnDA, and MeFOSAA, see Additional file 1: Fig. S5). There were some CpG sites that overlapped between 2 PFAS, but no CpG was universally associated with all PFAS (Fig. 4A). Similarly, there were several genes with significant differences in 5-hmC that were shared among PFAS, but no single gene was shared among all PFAS (Fig. 4B). Hydroxymethylation in three genes was associated with at least five of the seven PFAS (see Fig. 4C), including *SHANK2* (SH3 and multiple ankyrin repeat domains 2), *PARD3* (par-3 family cell polarity regulator), and *MYH9* (myosin heavy chain 9). There were 88 other genes that had differences in hydroxymethylation associated with four PFAS, with the majority (73 of 88) shared between PFOS, PFNA, PFDA, and PFUnDA (Fig. 4B, Additional file 2: Table S4). For individual PFAS, sites with differences in 5-hmC were similarly distributed across gene locations (Additional file 1: Fig. S6), but there were some discrepancies in the distribution of sites in relation to CpG islands (Additional file 1: Fig. S7), with proportionately fewer significant 5-hmC sites in actual CpG islands, when compared to all sites included on the EPIC array.

Regional differences in 5-hmC were identified using results from hydroxymethylation models for each PFAS associated with at least 100 sites. PFDA was the only PFAS with significant regions (*q* < 0.05). Top regional differences are reported in Table 5.

**Table 5:** Top regional 5-hmC differences related to PFDA exposure

Chromosome	Position start	Position end	*p*-value	BH *q*−value	Number probes	Gene name
1	47780564	47783925	8.74E−07	6.92E−04	4	*TRABD2B*
6	97600110	97602965	2.82E−06	9.56E−04	4	*ENSG00000271860.9*
1	9775829	9776507	2.50E−07	5.24E−04	3	*CLSTN1*
12	78682326	78683267	4.41E−07	5.24E−04	3	
4	88736370	88738396	1.60E−06	7.20E−04	3	*FAM13A*
7	9787186	9789187	4.58E−06	0.0012	3	
6	79762944	79765119	1.09E−05	0.002	3	
1	7614319	7617563	1.53E−05	0.002	3	*CAMTA1*
6	19619236	19620188	1.54E−05	0.002	3	*LNC-LBCS*
4	12758255	12761141	1.58E−05	0.002	3	
6	95632160	95632937	1.64E−05	0.002	3	
5	84773408	84774104	1.78E−05	0.002	3	
2	93797164	93798143	2.18E−05	0.0022	3	
4	9649184	9650927	3.28E−05	0.0024	3	*ENSG00000287117.1*
7	99653239	99655923	3.68E−05	0.0024	3	*CYP3A5*
7	73708261	73711118	5.92E−05	0.0028	3	*STX1A*
8	72631834	72631937	1.19E−04	0.0033	3	*KCNB2*
1	82669840	82672849	4.25E−04	0.0049	3	*ENSG00000233290.4*
3	32696525	32699280	6.69E−04	0.0055	3	*CNOT10*
7	4697496	4698880	7.72E−04	0.0058	3	*FOXK1*

Describes the chromosomal locations of significant regions of 5-hmC related to PFDA exposures. *p* represents the uncorrected *p*-value, and *q* represents the Benjamini–Hochberg (BH) corrected *q*-value

KEGG pathway analysis was conducted using results from hydroxymethylation models for each PFAS associated with at least 100 sites. While no pathway met a *q* < 0.05 cutoff for enrichment, each PFAS had several pathways that were of interest (*p* < 0.05 with at least 2 differentially hydroxymethylated genes in the pathway, Additional file 1: Table S7). Within this analysis, there were several overlapping functions and specific pathways that were associated with differential hydroxymethylated genes across many PFAS. In general, the most common classifications that were associated with hydroxymethylation differences with any PFAS were in neuroendocrine system pathways. Within specific KEGG pathways, the glutamatergic synapse pathway was enriched among genes associated with PFHxS and PFOS; Huntington disease was associated with genes from the PFOS and PFNA models; insulin secretion, gonadotropin-releasing hormone (GnRH) secretion, and high-affinity IgE receptor (FcεRI) signaling was enriched among genes from the PFNA and PFDA models (*p* < 0.05).

### PFAS and birth outcomes: mediation by epigenetics

Across all families with passing total DNA methylation data (*n* = 141), there were several relationships suggestive of significance (*p* < 0.1) between birth outcomes (gestational age at birth, or Fenton z-scores) and PFAS exposures, when controlling for the necessary confounders for statistical mediation analyses (Table 6). When controlling for parity, self-reported race, and smoking status, concentrations of PFHxS were related to decreased Fenton z-score (*β* = −0.25, *p* = 0.036). There were also negative relationships of both PFNA (*β* = −0.31, *p* = 0.089) and PFUnDA (*β* = −0.46, *p* = 0.019) with gestational age (measured in weeks).

**Table 6:** Associations between PFAS and birth outcomes (n = 141)

PFAS	Gestational age (weeks)	Fenton Z-score: size-for-gestational age, corrected by sex
PFHxS	*β* = 0.04 ± 0.21 *p* = 0.85	*β* = −0.25 ± 0.16 *p* = 0.036
PFOS	*β* = −0.160 ± 0.19 *p* = 0.40	*β* = −0.12 ± 0.15 *p* = 0.41
PFOA	*β* = −0.056 ± 0.17 *p* = 0.75	*β* = −0.036 ± 0.14 *p* = 0.79
PFNA	*β* = −0.31 ± 0.19 *p* = 0.089	*β* = −0.016 ± 0.15 *p* = 0.91
PFDA	*β* = −0.27 ± 0.17 *p* = 0.114	*β* = 0.13 ± 0.13 *p* = 0.383
PFUnDA	*β* = −0.46 ± 0.19 *p* = 0.019	*β* = 0.010 ± 0.16 *p* = 0.95
MeFOSAA	*β* = 0.15 ± 0.20 *p* = 0.45	*β* = −0.059 ± 0.16 *p* = 0.71

Relationships with a *p*-value < 0.1 were considered for mediation

MeFOSAA—2-(N-methyl-perfluorooctane sulfonamido) acetic acid; PFAS—per-/polyfluoroalkyl substances; PFHxS—perfluorohexanesulphonic acid; PFDA—perfluorodecanoic acid; PFNA—perfluorononanoic acid; PFOA—perfluorooctanoic acid; PFOS—perfluorooctanesulfonic acid; PFUnDA—perfluoroundecanoic acid.

*Coefficients representing differences for each log-fold unit change in PFAS* concentration or categorical PFAS detection* and p-values from linear regressions assessing the relationship between PFAS and gestational age, birthweight, or Fenton z-score, using the following linear regressions:*

$${\text{Gestational}}\;{\text{Age}} = \beta _{0} + \beta _{1} {\text{PFAS}} + \beta _{2} {\text{Parity}} + \beta _{3} {\text{Race}} + \beta _{4} {\text{Smoking}}$$

$${\text{Fenton}}\;Z - {\text{Score}} = \beta _{0} + \beta _{1} {\text{PFAS}} + \beta _{2} {\text{Parity}} + \beta _{3} {\text{Race}} + \beta _{4} {\text{Smoking}}$$

To reduce the number of methylation sites assessed for mediation, only significant sites (*q* < 0.05) from total methylation, 5-mC, and 5-hmC analyses associated with the three birth outcome-associated PFAS (PFHxS, PFNA, and PFUnDA) were considered (Additional file 2: Table S6). For PFHxS, five 5-mC sites and six 5-hmC sites were related to Fenton z-score (*p* < 0.05) and selected for mediation analysis. For PNFA, two total methylation CpG sites, 37 5-mC sites, and 26 5-hmC sites were related to gestational age and selected for mediation. For PFUnDA, there were 60 sites for 5-mC and 57 sites for 5-hmC that were considered for mediation on gestational age.

For gene-wise sites as well as the collective group of sites within a methylation type and for an individual PFAS, kernel machine regression was used to assess the nonlinear, gene-wise mediation effects of methylation on the relationship between the PFAS and birth outcomes. After applying Benjamini-Hochberg (BH) corrections to the *p*-values, no single gene met a *q* < 0.05 for any exposure (Additional file 2: Table S7). Two genes with one CpG site each (*INADL*, or the PATJ crumbs cell polarity complex component, *q* = 0.07; *LOC100506023*, *q* = 0.07) were suggestive of a nonlinear mediation effect of 5-hmC between PFNA and gestational age. When assessing the total mediation of combined effects of all CpG sites included for each relationship (e.g., each PFAS, birth effect, and type of methylation), there were significant mediation effects of 37 5-mC sites and 26 5-hmC sites between PFNA exposure and gestational age (*q* = 1.28E−05 and *q* = 1.28E−05), as well as both 60 5-mC sites and 57 5-hmC sites between PFUnDA exposure and gestational age (*q* = 0.026 and *q* = 7.15E−04; see Table 7). Many of the genes these mediating CpG sites were in functions related to either cell proliferation and viability or apoptosis and cell death. Among potential mediators, there were several genes shared among the methylation types for PFNA (*VTI1B*, vesicle transport through interaction with T-SNAREs 1B, and *LOC100506023*) and PFUnDA (*RPIA*, ribose 5-phosphate isomerase A, *GTF3C2*, general transcription factor IIIC subunit 2, *SDK1*, sidekick cell adhesion molecule 1, *TLE3*, *TECPR2*, tectonin beta-propeller repeat containing 2, *ERN2*, endoplasmic reticulum to nucleus signaling 2, *LOC284395*). Similarly, there was one gene shared across exposures for 5-mC (*AKR7A3*, aldo–keto reductase family 7 member A3), and there were two genes shared for 5-hmC (*HEATR3*, HEAT repeat containing 3, and *GSDMA, g*asdermin A).

**Table 7:** Mediation by methylation across multiple loci

Exposure, mediator, and outcome	Number of CpG Sites	*p*-Value	Benjamini–Hochberg *q*-value
PFHxS, 5-mC, and Fenton-Z-Score	5	0.0393	0.136
PFHxS, 5-hmC, and Fenton-Z-Score	6	0.241	0.241
PFNA, Total methylation, and gestational age	2	0.00913	0.0738
PFNA, 5-mC, and gestational age	37	1.93E-07	1.28E-05
PFNA, 5-hmC, and gestational age	26	3.72E-07	1.28E-05
PFUnDA, 5-mC, and gestational age	60	4.57E-04	0.0263
PFUnDA, 5-hmC, and gestational age	57	6.22E-06	7.15E-04

Kernel machine regression results using all significant CpG sites meeting the following filter criteria: CpG related to PFAS exposure (*q* < 0.05), PFAS related to birth outcome (*p* < 0.1), and CpG related to birth outcome (*p* < 0.05). Individual gene-wise results are described in Additional file 2: Table S7. n = 141 for total methylation and n = 70 for 5-mC and 5-hmC

## Discussion

PFAS are widespread environmental contaminants that are actively impacting human health, with known effects on reproduction, immune and metabolic function, and development (for review, see Fenton et al. [48]). One mechanism that may underlie these effects is aberrant epigenetic programming, which has been observed in laboratory models and in human epidemiological cohorts. In our investigation of associations between PFAS and DNA methylation, we posited that any epigenetic differences may mediate the relationship of PFAS and adverse birth outcomes. Across our analyses, our hypothesis was largely supported; we found significant relationships between PFAS and DNA methylation (total, 5-mC, and 5-hmC), as well as PFAS and birth outcomes (decreased gestational age and Fenton z-scores for size at birth). Even more, we found a significant mediation effect of both 5-mC and 5-hmC (but not total methylation) on the relationship between both PFNA and PFUnDA and decreased gestational age at birth, demonstrating the mediation effects of not just general epigenetic differences, but specific types of DNA methylation marks, on the relationship between developmental PFAS exposure and birth outcomes.

These results strengthen the known evidence of the relationship of developmental PFAS exposure and early-life epigenetic differences [40–46], despite variations in both the study populations and PFAS exposure levels. One of the most frequently reported genes differentially methylated in epigenome-wide developmental studies of PFAS exposure is *TNXB* (tenascin-XB) [45, 46]. Presently, differences in only either 5-mC or 5-hmC, and not total methylation, were observed in this gene, suggesting that there are likely subtle differences in the type of methylation that could contribute to discordantly methylated genes observed across study populations. *TNXB* is also highly represented on Illumina arrays, which could contribute to commonalities that were previously reported [40]. These differences may be further exacerbated by sex-specific differences in epigenetics, which we were presently unable to assess in 5-mC or 5-hmC due to small sample sizes. Future studies should prioritize studies that are large enough to investigate these potentially important sex-specific effects in specific methylation types.

Presently and to the best of our knowledge, this is the hallmark study investigating prenatal PFAS exposure and 5-mC/5-hmC specifically. Results reported here show striking patterns of reductions in 5-hmC with concomitant increases in 5-mC, across six of the seven PFAS included. Differences in methylation that broadly occur across regions or within regulatory elements are more likely to be associated with gene expression changes [49, 50]. In a regional analysis of 5-hmC, we did find some genes that had broad regions of differential hydroxymethylation. These regions were often associated with regulatory elements, such as known gene enhancers or histone modifications. Compared to 5-mC, 5-hmC is proposed to be more closely linked to histone modifications and gene regulation [51]. Our data broadly supports this, but the EPIC array only covers loci in a small portion of known regulatory elements [52]. Follow-up with other methods, such as hydroxymethylated DNA immunoprecipitation sequencing (hMeDIP-Seq) or nano-hmC-Seal, could better investigate 5-hmC differences in important regulatory elements.

Some of these differences were also mediators of the relationship between PFAS and decreased gestational age, with trends towards mediating PFAS-related decreases in size at birth. These two birth outcomes may be early indicators of adverse neurocognitive [53, 54] and behavioral/emotional [55] effects later in life. In the brain, 5-hmC is thought to modulate mammalian postnatal neurodevelopment, with marked increases from early postnatal stages to adulthood [56]. Pathway analyses of 5-hmC genes suggested that there were differences in the status of genes related to general cell processes, as well as functions in the endocrine, immune, and nervous systems. While the evidence of the cognitive effects of prenatal PFAS exposure is presently mixed [57–61], other health effects resulting from gestational PFAS exposure are well documented [10, 19, 20, 48]. Given enrichment of pathways relevant to neurological function, the endocrine system, and insulin secretion among the PFAS-associated genes, the role of 5-hmC in the development of long-term adverse health outcomes is an essential area for future investigation.

PFAS may disrupt epigenetic programming through the widespread dysregulation of epigenetic machinery and/or other PFAS mechanisms of toxicity. A primary mechanism of interest is PFAS-induced oxidative stress [20], which has been widely documented in vitro [62–65] and in human epidemiological cohorts [66–68]. While some researchers have suggested that this relationship could lead to genotoxicity or cytotoxicity [63], others have observed PFAS-induced oxidative stress without any evidence of either of these effects [64]. Seminal work with mice and cells demonstrated that oxidative stress may directly alter TET enzymes that are responsible for the formation of 5-hmC, leading to widespread decreases in 5-hmC across the genome [51]. Changing TET activity may be a compensatory mechanism to combat the deleterious effects of oxidative stress that may also be connected with alterations in the hydroxymethylation of noncoding RNAs that could contribute to epigenetic regulation. Interestingly, many other researchers have noted differences in noncoding RNAs that were related to PFAS exposure [69–72]. While we did not investigate these other epigenetic regulators, there were many genes encoding noncoding RNAs with significant associations between PFAS and 5-hmC. This complex interplay of epigenetic machinery, oxidative stress, and endpoints (i.e., 5-hmC) is an essential area to understand the molecular mechanisms underpinning toxicity by environmental contaminants.

Overall, results in this manuscript detail a compelling and complex interplay of early-life exposures to PFAS, specific differences in DNA methylation types, and adverse birth outcomes. The link to birth outcomes is particularly important for public health, as this cohort represents a group of mothers and infants who were healthy at birth (full term, no known complications). Because cases of extreme preterm birth or other severe birth outcomes were excluded from the cohort, our results indicate that there are subtle, but important relationships between PFAS, epigenetics, and birth outcomes. Exploration was limited by small sample sizes available for these measures, as well as the time points and tissue types available for assessment. One time point of particular interest to the present study is that of exposure; PFAS were measured in blood samples from early maternal pregnancy which is known to have levels much higher than fetal tissues [73]. These levels, however, remain consistent and/or increase in fetal tissues over time. Because we were measuring DNA modifications that are rewritten in the early embryonic stages, additional research is needed to clarify the maternal–fetal kinetics of these PFAS and the relationship of kinetics with epigenetic differences and birth outcomes.

Additionally, while we were able to delineate PFAS-related effects between specific methylation types (5-mC and 5-hmC), we were unable to assess the sex-specific effects in these markers. Our methods also selected for the probes with higher levels of 5-hmC and those that had an interaction term between 5-mC and 5-hmC, which could be contributing to the large number of significant sites observed in the separate 5-mC and 5-hmC analyses. As total methylation related to PFAS has sex-specific differences and the Illumina EPIC array selects for sites that may not best represent important environmentally induced changes [74], additional work should prioritize large enough sample sizes and appropriate methods needed to confirm these results. Due to the small sample size, we also selected for other precision variables, such as cell types. Research with larger sample sizes that allow for the inclusion of other important precision variables is warranted. In this manuscript, we were able to assess seven unique PFAS, but thousands more exist that humans may be exposed to. There has been some speculation that different PFAS moieties may have varying mechanisms of action, additive/multiplicative effects, or cumulating effects that should be considered [75, 76]. Finally, while there has been evidence of total methylation differences related to PFAS exposures across many types of cohorts [41–46], our cohort was rather demographically homogeneous and did not capture the full implications of health disparities caused by racism and structural policies. In particular, we found that PFAS exposure was only associated with self-reported race as Black or African American, which likely does not capture all structural factors that affect public health and birth outcomes. Going forward, research should continue to expand on the significant findings here, while also addressing these limitations, to best understand the health effects from these important environmental contaminants.

This is the first report to our knowledge of widespread 5-hmC alterations by prenatal PFAS exposure. These results were observed in a healthy birth cohort with modest PFAS exposures, suggesting that the developmental epigenetic impacts from PFAS may be sufficiently concerning in the general population, especially in populations with higher exposure burdens. As we continue to develop our understanding of PFAS, the long-term impacts of the demonstrated relationship should be carefully considered.

## Materials and methods

### Study population and sample collection

The MMIP is a birth cohort study based out of the University of Michigan Von Voigtlander Women’s Hospital, which recruited participants from 2010 to 2019 [77, 78]. Participants were recruited during their first prenatal visit if they were at least 18 years old, had a singleton pregnancy, were between 8 and 14 weeks gestation, and intended to deliver at the University of Michigan Hospital. All participants provided informed, written consent prior to study enrollment. The University of Michigan Medical School Institutional Review Board approved all study procedures (Approval no. HUM00017941).

Venous blood samples were collected during the first trimester prenatal visit by trained phlebotomists. Blood samples were collected in BD Vacutainer EDTA-preserved tubes (Becton, Dickinson and Company) and were centrifuged at 1000 relative centrifugal force for 10 min. Separated plasma was then stored at -80 ºC until analysis. Shortly after delivery, cord blood was collected via standard venipuncture into EDTA-preserved tubes (Becton, Dickinson and Company) and PaxGene DNA and RNA tubes (PreAnalytix). EDTA tubes were centrifuged, and plasma was aliquoted into tubes. Plasma, DNA tubes, and RNA tubes were frozen at -80 ºC until further processing. At that time, samples were selected for laboratory analysis, 309 participants had been recruited during the first trimester, of which 288 had remained in the study and given birth at the University of Michigan Hospital; 206 of these participants that had an adequate volume of first trimester plasma collected and were selected for PFAS analysis. Among these, DNA methylation was completed on those with cord blood samples preserved for DNA isolation. Those that passed stringent quality control (see below, *DNA Methylation Processing*, *n* = 141) were included (see study schematic in Fig. 1).

### Survey and birth outcome data

At the first trimester visit, maternal weight and BMI were ascertained from the medical records and a survey was administered to collect information on demographics, including race, ethnicity, age, parity, marital status, and smoking status. For analysis, smoking status was considered as binary of either smoking reported in pregnancy or not. Race was collected as self-described racial and ethnic identities. For analysis, race was included as proxy for racism and racist policies that are related to health. Parity was considered as a numerical variable.

At delivery, data were collected from the health records on infant sex, gestational age, and birth anthropometry. Gestational age was recorded as the healthcare provider’s best estimate from either the last menstrual period or ultrasound, as recommended by the American Congress of Obstetricians and Gynecologists. Fenton z-scores were calculated from infant sex, birthweight, and gestational age using the PediTools website (https://peditools.org/fenton2013/ [79]).

### PFAS analysis

Concentrations of nine PFAS were quantified in first trimester maternal plasma samples through the NSF International laboratory (Ann Arbor, MI). The measured PFAS were: 2-(N-methyl-perfluorooctane sulfonamido) acetic acid (MeFOSAA), perfluorooctanesulfonamide (PFOSA), perfluorohexanesulphonic acid (PFHxS), perluoroheptanoic acid (PFHpA), perfluorooctanoic acid (PFOA), perfluorooctanesulfonic acid (PFOS), perfluorodecanoic acid (PFDA), perfluorononanoic acid (PFNA), and perfluoroundecanoic acid (PFUnDA). Concentrations were measured via a method based off the US Centers for Disease Control and Prevention (CDC) Polyfluoroalkyl Chemicals Method Laboratory Procedure 6304.1 [80]. This method uses on-line solid phase extraction coupled with high-performance liquid chromatography–isotope dilution tandem mass spectrometry. Analysis was performed using a Thermo Scientific Transcend TXII Turbulent Flow system (ThermoFisher Scientific) interfaced with Thermo Scientific Quantiva triple quadrupole mass spectrometer (ThermoFisher Scientific) using MRM in negative mode. The method incorporates calibration curve checks and known standards interspersed with study samples to ensure accuracy and precision. The limits of detection (LOD) were established by replicate injections of low concentration standards (Additional file 1: Table S1). The laboratory was part of the National Institute of Health Children’s Health Exposure Analysis Resource network (NIH CHEAR) at the time and participated in inter-lab quality control and quality assurance [81].

### DNA methylation analysis

DNA was isolated from nucleated cells of cord blood (leukocytes and nucleated red blood cells) via a Paxgene DNA isolation kit (PreAnalytix) according to the manufacturer’s protocol. DNA concentrations were quantified via the Quant-IT Picogreen double stranded DNA assay (ThermoFisher Scientific). DNA methylation profiles were assessed with the Infinium MethylationEPIC BeadChip (Illumina), which covers over 850,000 unique methylation sites (CpG sites) [82]. DNA was bisulfite converted using the EZ DNA Methylation kit (Zymo Research) and assayed on the BeadChip. As bisulfite conversion quantifies total methylation and cannot distinguish between 5-mC and 5-hmC, this traditional analysis can be considered ‘total methylation’ (5-mc + 5hmC). To profile 5-mC and 5-hmC individually in a subset of samples (n = 90 before quality control), parallel bisulfite conversion and oxidative bisulfite conversion was performed using the Nugen TrueMethyl oxBS Module (NuGEN Technologies, Inc.). In this protocol, samples are oxidized, converting 5-hmC to 5- formylcytosine (5-fC), which is then converted to uracil following bisulfite treatment, leaving only 5-mC as cytosine residues. Following both bisulfite and oxidative bisulfite treatments, samples were randomized across chips and chip positions, hybridized to BeadChips, and signals were read at the University of Michigan Advanced Genomics Core.

For samples with traditional bisulfite conversion, the final data consist of average betas representing the proportion of total methylated cytosine (5-mC + 5-hmC) for each site. For sample aliquots undergoing oxidative treatment, final data consist of betas representing the proportion of 5-mC only. This procedure has been used to generate 5-hmC data from BeadChips by other epidemiological studies [83–85]. Instead of simply subtracting 5-mC from total DNA methylation at each CpG site to estimate 5-hmC levels (which can result in negative values in hypomethylated sites), the Maximum Likelihood Methylation Levels (MLML) method, available in the R package MLML2R, was used to estimate 5-hmC [86, 87]. The computationally efficient MLML method accepts data from bisulfite sequencing or Infinium arrays and simultaneously estimates 5-mC, 5-hmC, and 5-C (unmethylated) proportions at each loci using an algorithm that does not allow negatives or summed scores over 1 [87]. Prior to performing MLML, data were preprocessed together as described below, with the exception of quantile normalization. Estimated 5-hmC and 5-mC from the MLML method were used in analyses.

### DNA methylation processing

Quality control (QC) and preprocessing (i.e., normalization) of the array data following both treatments was completed in R (version > 4.1) using Bioconductor packages (minfi, Enmix) [88–92]. Briefly, raw image IDAT files for all samples were read into R. In individual IDAT files for each treated DNA sample, samples with poor coverage (< 3 beads), samples with > 5% of sites failed, and samples of which predicted sex did not match reported sex and/or genetic background did not match that of matching maternal samples were removed from the dataset. In either the bisulfite treated group or the oxidative bisulfite group, probes with poor detection (*p* < 1e^−16^ when compared with background) were removed. After these steps, 31,434 CpG sites and 39 samples were removed from all datasets. Additional probes with SNPs in the CpG or single base extension site, probes known to be cross-reactive [52, 93], CH probes, and probes in the X/Y chromosomes were removed. Probes that had high intra-sample variability (> 5% difference, based on 20 replicated samples) were also removed. The same probes were removed from both bisulfite treated and oxidative bisulfite treated samples, yielding 744,926 high-quality probes in all datasets. For replicated samples, one sample that passed all QC checks was randomly selected for inclusion in analysis. For those passing QC, correction and normalization was completed on each group (bisulfite data and oxidative bisulfite data describing both 5-hmC and 5-mC). Background correction with out-of-band (oob) and dye bias correction with RELIC were applied [89]. Quantile normalization was applied on each probe type and color channel separately [90].

Cell type proportions were estimated using an algorithm based on a reference dataset from seven sorted cord blood cell types [94, 95]. Surrogate variable analysis was performed on data from the control probes to create variables that best estimate the technical variability of samples [96]. For total methylation, 141 unique MMIP samples with PFAS data passed QC. Out of these, 70 also had a matching oxidative bisulfite converted sample that passed QC.

### Statistical analysis

Analyses were performed in R, version > 4.1 [97]. PFAS plasma concentration distributions were evaluated for normality and any potential outliers. Individual concentrations below the analytical limit of detection (LOD, see Additional file 1: Table S1) were replaced with a value of $LOD/\sqrt{2}$. The LOD for all PFAS was 0.1 µg/L. Any PFAS with > 80% of samples below the LOD were not included in any further statistical analyses (PFOSA and PFHpA). PFAS with 40–80% of samples below the LOD were dichotomized (above the LOD vs. below the LOD) and treated as a categorical variable in subsequent analyses (PFUnDA and MeFOSAA). PFAS that had < 40% below the LOD remaining were treated as continuous variables (PFHxS, PFOA, PFOS, PFNA, and PFDA), consistent with recommended handling of PFAS data by NHANES [98]. PFAS concentrations for these variables were natural log-transformed prior to analysis. Correlations of all seven PFAS included in this analysis were examined to determine the relationship between the exposures of interest.

**Fig. 5:**
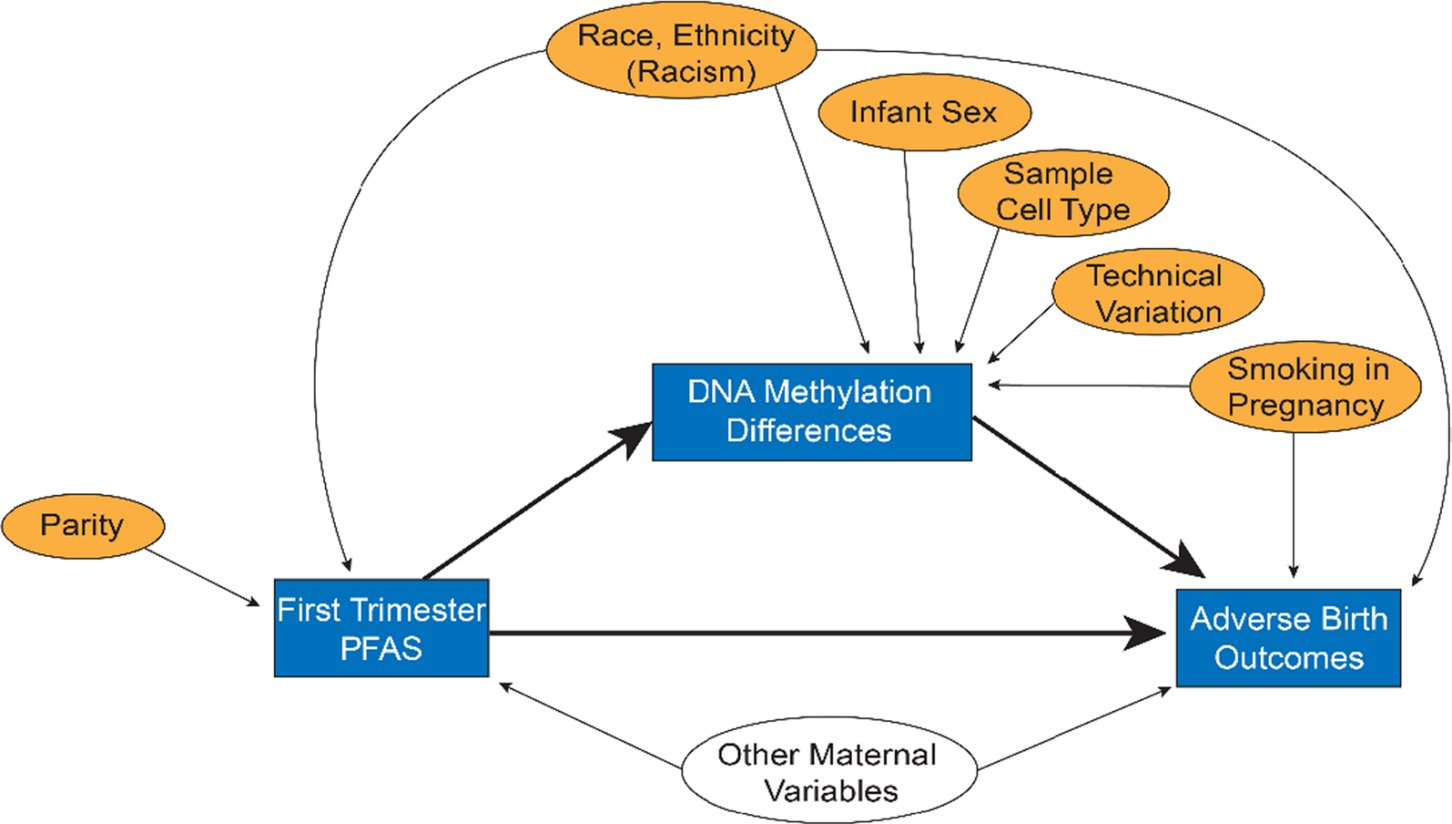
A directed acyclic graph (DAG) used to identify real and theoretical confounders in the relationship between PFAS exposure and newborn DNA methylation and hydroxymethylation. Rectangles represent variables included as exposures or outcomes. Ovals represent confounding or control variables that were assessed for model inclusion. Thick solid lines show the relationship considered for mediation analysis. Filled ovals represent all precision, confounding, or control variables included in the models. Other maternal variables (unfilled oval on bottom of DAG) were considered for inclusion (age, early pregnancy body mass index (BMI), marital status, and income). Within our dataset, only one variable (self-reported race as a proxy for racism and racist policies) showed evidence as a true confounder (associated with both exposure and outcome); other maternal variables were not included in the models. Infant sex was not considered as true confounder, as only gestational age and Fenton z-scores (already adjusted for age and sex) were assessed as outcomes

Descriptive statistics were computed for all continuous and categorical variables, and differences between cohort groups (entire cohort, those with passing total methylation, and those with passing 5-mC/5-hmC data) were compared using one-way ANOVAs. A directed acyclic graph (DAG) was constructed to identify confounders of interest in the relationship between PFAS and DNA methylation (Fig. 5). Infant sex, gestational age, and maternal characteristics (early pregnancy BMI, age, parity, race and ethnicity, smoking status, marital status) were initially considered as potential confounders, and correlations of these confounders with PFAS were assessed using either Pearson’s correlations for numeric variables or Chi-squared tests for categorical variables. Technical variables including estimated cord blood cell type proportions and batch variables were the main predictors of variability within the DNA methylation and DNA hydroxymethylation data, as determined by surrogate variable analysis in the R package ChAMP [99, 100].

To minimize confounding bias and the impact of cell-type and batch effects while not overfitting methylation data, ten variables were selected for inclusion in the final model. Infant sex, parity, smoking status, and self-reported race as African American or Black (as a proxy of racism and racist policies that influence PFAS exposure burden) were significantly associated with at least one PFAS (*p* < 0.05) and considered true confounders in this study population in the main relationship of interest (association with both PFAS and DNA methylation or hydroxymethylation). The top three cell type proportions that were significantly associated with at least one PFAS were selected for model inclusion (CD4 + T cells, CD8 + T cells, and granulated cells). Nucleated red blood cells were also selected for model inclusion, as this type of cell is uniquely common in neonatal blood, and may significantly impact the methylation profiles in cord blood [101]. Finally, the top two component variables (PCs from the surrogate variable analysis described above) to account for batch effects were included in the models.

For all models, methylation was regressed on individual PFAS exposures, as continuous PFAS concentrations (natural log transformed PFHxS, PFOA, PFOS, PFNA, PFDA) or categorical PFAS (above the LOD and below the LOD for PFUnDA and MeFOSAA). Any outliers for continuous measures of PFAS were kept in analyses and believed to represent real exposure data. For all analyses, beta regressions, which are designed to explicitly model continuous proportional data, were fit using normalized beta values which represent the proportion of methylation at each CpG site (between 0 and 1). The GAMLSS R package [102] was used to regress beta values at each CpG site on the individual PFAS, adjusting for parity (numeric; 1–4), reported smoking during pregnancy (any versus none); self-described race as African American or Black (versus any other); infant sex, estimated cell type proportions for granulocytes, CD4 + T, CD8 + T, and nucleated red blood cells; and PC1 and PC2 representing technical/batch effects. Data for parity, infant sex, cell types, and PCs were available in all participants. Nine individuals were missing smoking status and six individuals were missing race. Missingness in these categories was found to be unrelated to exposures, so an imputation method was applied. In brief, the distributions of the complete data were defined, and random samples were drawn from these distributions. This method utilizes the first step of multiple imputation, but it does not run the analysis multiple times (due to the extreme computational load required for this analysis). In this case, sampled missing variables were imputed with the majority category by chance. Model inflation was assessed using genomic inflation factors (lambdas), comparing all raw *p*-values from each model to an expected distribution. Results from each model were considered after applying a BH procedure. CpG sites were annotated with data available from the IlluminaHumanMethylationEPICanno.ilm10b2.hg19 R package.

#### PFAS and total DNA methylation

For total DNA methylation (*n* = 141), the relationship between each PFAS with 744,926 loci were assessed using beta regressions as outlined above. Results were considered to be statistically significant with a BH false discovery rate (FDR) cut-off of *q* = 0.05. To examine potential sex-specific effects, additional models were run including an interaction term for *sex x PFAS*. CpG sites with significant interaction terms at a BH-corrected q-value of 0.05 were then stratified by sex.

#### PFAS and DNA hydroxymethylation

For 5-mC and 5-hmC (*n* = 70), an approach proposed by Kochmanski et al. [103] was used. Because hydroxymethylation is biologically and methodologically linked with methylation, interdependence precludes independently modeling these values. Instead, paired data can be evaluated to assess site level differences in methylation and hydroxymethylation. Estimated 5-hmC and 5-mC data from the MLML method were concatenated into a single matrix, resulting in two observations for each individual, with replicated phenotype data. An additional term (Type) was added to delineate if an observation was 5-mC or 5-hmC data. Because 5-hmC does not uniquely occur throughout the genome, any CpG site with a total (5-mC + 5-hmC) methylation of < 0.1 was excluded from analysis, yielding a total of 528,389 sites. For these 140 observations from 70 mother–infant pairs, associations with PFAS were tested using beta regressions in GAMLSS, with a random-effect for the ID, and allowing the methylation type to have independent Φ (identity link functions):

$$\begin{aligned} 5 - mC\;{\text{or}}\;5 - hmC\;{\text{proportion}} & = \beta _{0} + \beta _{1} {\text{PFAS}} + \beta _{2} {\text{Parity}} + \beta _{3} {\text{Smoking}} + \beta _{4} {\text{Race}} + \beta _{5} {\text{sex}} \\ & \quad + \beta _{6} {\text{CD}}4{\text{T}} + \beta _{7} {\text{CD}}8{\text{T}} + \beta _{8} {\text{GranCell}} + \beta _{9} n{\text{RBC}} \\ & \quad + \beta _{{10}} {\text{PC}}1 + \beta _{{11}} {\text{PC}}2 + \beta _{{12}} {\text{Type}} \\ & \quad + \beta _{{13}} {\text{Type}}*{\text{PFAS}} + [1|{\text{ID}}] \\ \end{aligned}$$

CpG sites with a BH-corrected interaction term of with *q*-value < 0.2 were than stratified by methylation type; associations between PFAS with 5-mC and 5-hmC were then modeled separately at these loci. Genomic inflation values were calculated to assess potential *p*-value inflation. A significance cutoff of q-value < 0.2 was used at this stage to reduce the number of tests and limit the number of sites with separate 5-mC and 5-hmC modeling. Within only these sites, a beta regression model identical to the model for total methylation (above) was fit for either 5-mC or 5-hmC. A q-value < 0.05 was used to identify either 5-mC or 5-hmC sites that were significantly associated with the PFAS of interest. Sex-stratified analyses were not included in this analysis, as the sample size for 5-mC and 5-hmC specific data was limited.

#### Posthoc methylation assessments

To better understand the public health implications of any significant relationships between PFAS, total methylation, 5-mC and 5-hmC, several post hoc assessments were conducted. For the total methylation analysis without sex-stratification, a correlation analysis was used to compare the directionality of the coefficients to results from any previously reported study that examined PFAS exposure and genome-wide total methylation differences in early life [41–46]. Sex-stratified results were not compared. For models with more than 1000 significant sites (in 5-hmC analyses), regional differences were assessed using ipDMR [104], using 310 bp bins. For models with more than 100 significant sites (in 5-hmC analyses), KEGG pathways were assessed using the methylGSA package in R.

#### Epigenetic mediation assessment

Because birth outcomes have previously been associated with PFAS exposure, differences in DNA methylation were considered as potential mediators in the exposure to outcome pathway. To assure assumptions for mediation analyses were met in this study population [105], the direct relationship between PFAS exposure and birth outcomes, including gestational age and Fenton z-score adjusted size-for-gestational age at birth was computed in R using linear regressions that controlled for parity, race and smoking status in pregnancy.

Mediation analyses were conducted using a nonlinear, kernel machine regression, which was specifically designed for epigenetic studies [106, 107]. To meet the assumptions for mediation, only those relationships with effects suggestive of significance were considered for mediation. Relationships were screened first at *q* < 0.05 for the association between PFAS and any type of methylation; then *p* < 0.1 for PFAS and birth outcomes; and finally, *p* < 0.05 for any type of methylation and birth outcomes. Gene-wise CpG sites meeting these criteria were included as mediators. Additionally, the complete groups of all sites related to any single PFAS and birth outcome were also assessed. BH q-values were applied to all comparisons by PFAS, and a q-value < 0.05 was considered as statistically significant. Significant genes’ functions were identified using the human causal genes in Ingenuity Pathway Analysis’s BioProfiler tool (Qiagen).

## Supplementary Information

**Additional file 1.** Supplemental Tables and Figures

**Additional file 2.** Supplemental Excel Materials.

## Data Availability

Data from the MMIP study are available through the National Institutes of Health Human Health Exposure Analysis Resource (NIH HHEAR) data repository (dois: 10.36043/2273_357, 10.36043/2273_358, 10.36043/2273_338, and 10.36043/2273_337).

## Abbreviations

5-hmC: 5-Hydroxymethylcytosine
5-mC: 5-Methylcytosine
BH: Benjamini–Hochberg
BLOD: Below the limit of detection
BMI: Body mass index
CD4T: CD4 + T lymphocytes
CD8T: CD8 + T lymphocytes
CDC: Centers for Disease Control and Prevention
CpG: Cytosine-guanine sites
DAG: Directed acyclic graphic
FDR: False discovery rate
GranCell: Granulated cells
LOD: Limit of detection
MeFOSAA: 2-(N-methyl-perfluorooctane sulfonamido) acetic acid
MLML: Maximum Likelihood Methylation Levels
MMIP: Michigan Mother Infant Pairs
nRBC: Nucleated red blood cells
PC: Principal component representing batch effects
PFAS: Per-/polyfluoroalkyl substances
PFHpA: Perfluoroheptanoic acid
PFHxS: Perfluorohexanesulphonic acid
PFDA: Perfluorodecanoic acid
PFNA: Perfluorononanoic acid
PFOA: Perfluorooctanoic acid
PFOS: Perfluorooctanesulfonic acid
PFOSA: Perfluorooctanesulfonamide
PFUnDA: Perfluoroundecanoic acid
QC: Quality control
TET: Ten-eleven translocation
