# Supplementary material for: Mediation effects of DNA methylation and hydroxymethylation on birth outcomes after prenatal per- and polyfluoroalkyl substances (PFAS) exposure in the Michigan mother–infant Pairs cohort
Source: Clin Epigenetics. 2023 Mar 24;15:49. doi: 10.1186/s13148-023-01461-5 (PMC10037903; doi:10.1186/s13148-023-01461-5)
Supplement: Supplementary file 1 — Additional file 1. Supplemental Tables and Figures [file 13148_2023_1461_MOESM1_ESM.docx]

**Table S1: Plasma PFAS Concentrations (n=141)**

| PFAS | LOD (µg/L) | Count Above LOD (n=141) | >LOD | <LOD | Analysis Plan | GM  (µg/L) | GSD | Mean | Min | Max |
| --- | --- | --- | --- | --- | --- | --- | --- | --- | --- | --- |
| PFHxS | 0.1 | 141 | 100.00% | 0.00% | Numeric | 3.191 | 1.61 | 3.597 | 0.976 | 21.766 |
| PFOS | 0.1 | 141 | 100.00% | 0.00% | Numeric | 5.253 | 1.74 | 6.014 | 0.625 | 22.256 |
| PFOA | 0.1 | 126 | 89.36% | 10.64% | Numeric | 1.142 | 1.88 | 1.373 | <LOD | 4.13 |
| PFNA | 0.1 | 139 | 98.58% | 1.42% | Numeric | 0.365 | 1.75 | 0.421 | <LOD | 1.263 |
| PFDA | 0.1 | 84 | 59.57% | 40.43% | Numeric | 0.124 | 1.77 | 0.148 | <LOD | 0.583 |
| PFUnDA | 0.1 | 50 | 35.46% | 64.54% | Dichotomize | 0.106 | 1.87 | 0.138 | <LOD | 0.988 |
| MeFOSAA | 0.1 | 53 | 37.59% | 62.41% | Dichotomize | 0.107 | 1.96 | 0.155 | <LOD | 2.608 |

*Shows the limit of detection (LOD), number and percentage of the samples above or below the LOD, how the PFAS were included in the analysis (based on a detection rate of at least a rounded detection rate of 60% above the LOD). Any sample below the LOD was imputed with* ${LOD}/\sqrt{2}$*. The geometric mean (GM), geometric standard deviation (GSD), means, and the minimum (Min) and maximum (Max) of the PFAS concentrations in first-trimester maternal plasma are reported in the right-hand side of the table. All values are reported in untransformed values. PFHpA (perfluoroheptanoic acid) and PFOSA (perfluorooctanesulfonamide) were also measured but excluded from analysis due to poor detection (<80% below the LOD). Abbreviations: MeFOSAA – 2-(N-methyl-perfluorooctane sulfonamido) acetic acid; PFAS – per-/polyfluoroalkyl substances; PFHxS – perfluorohexanesulphonic acid; PFDA – perfluorodecanoic acid; PFNA – perfluorononanoic acid; PFOA – perfluorooctanoic acid; PFOS – perfluorooctanesulfonic acid; PFUnDA – perfluoroundecanoic acid.*

*
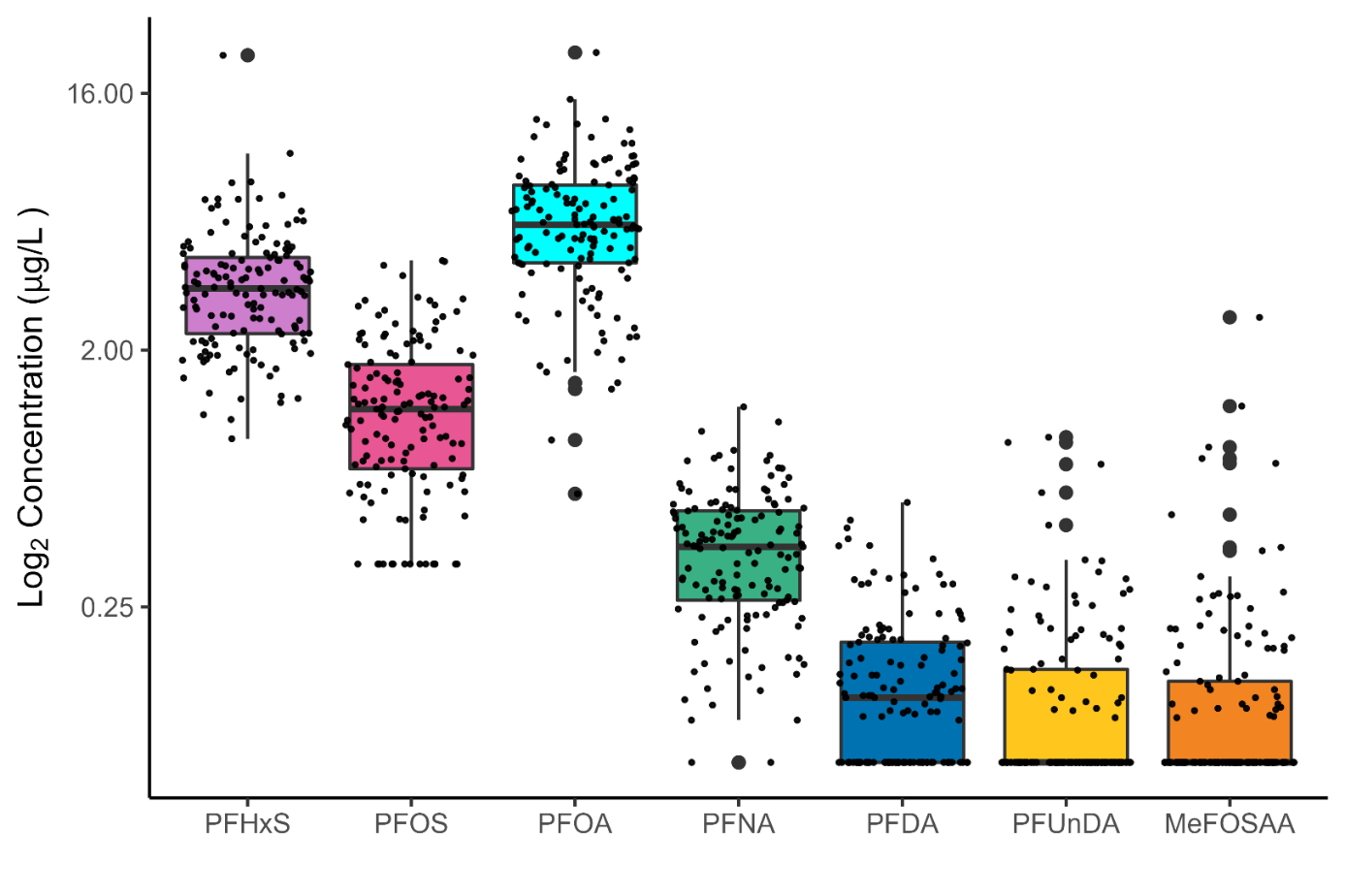
*

**Fig. S2: Natural log concentrations (****µg/L) of PFAS in maternal plasma in the first trimester of pregnancy.** Each point overlayed on the box plot represents an individual measurement (n=141). PFHxS, PFOS, PFOA, PFNA, and PFDA had at least 60% of samples above the limit of detection and were analyzed as continuous variables (Table S1). PFUnDA and MeFOSAA had less than 60% of samples of the limit of detection and were analyzed as either detected or not (Table S1). Abbreviations: MeFOSAA – 2-(N-methyl-perfluorooctane sulfonamido) acetic acid; PFAS – per-/polyfluoroalkyl substances; PFHxS – perfluorohexanesulphonic acid; PFDA – perfluorodecanoic acid; PFNA – perfluorononanoic acid; PFOA – perfluorooctanoic acid; PFOS – perfluorooctanesulfonic acid; PFUnDA – perfluoroundecanoic acid.


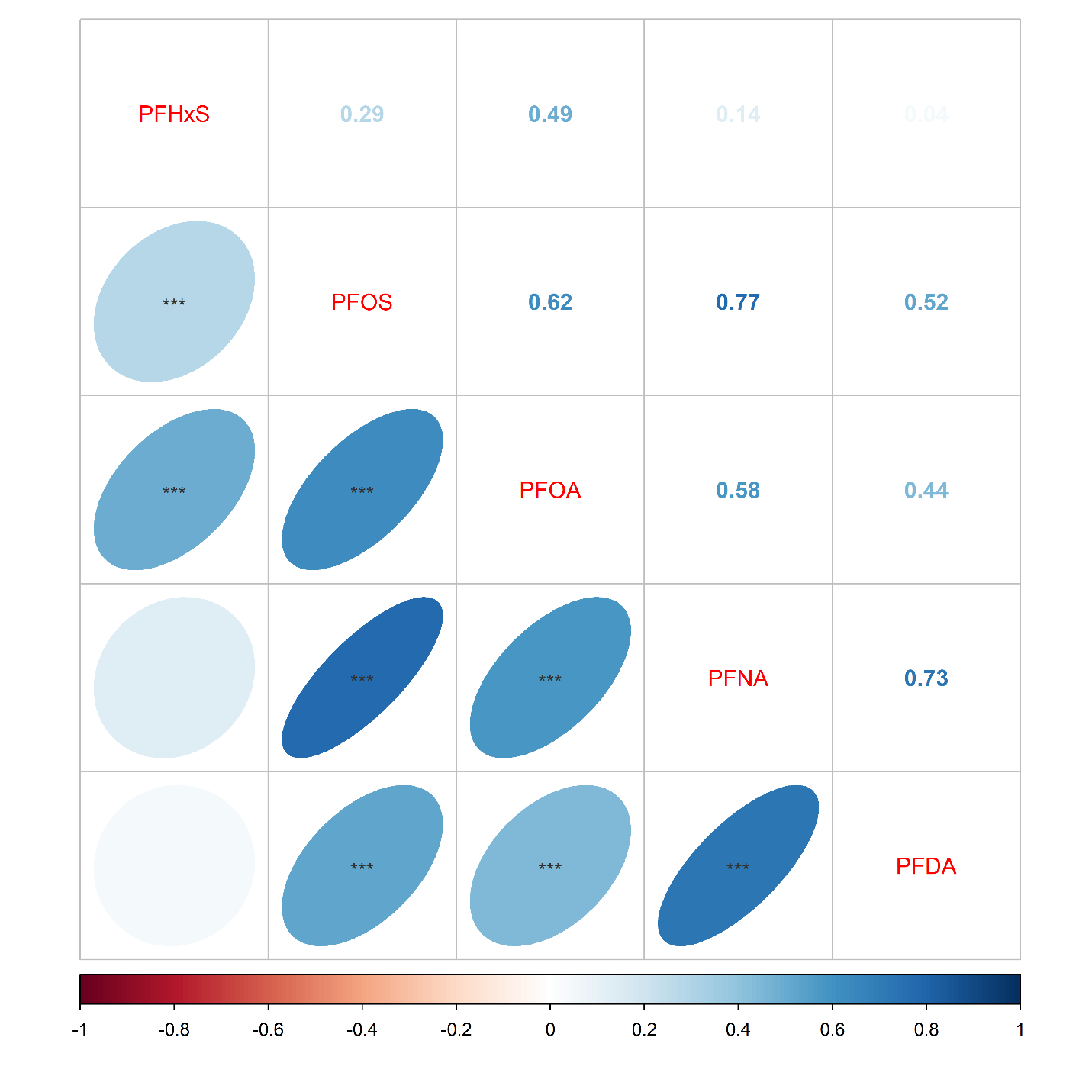


**Fig. S3: Pearson’s correlation plot of PFAS concentrations in maternal plasma in the first trimester of pregnancy**. Numbers represent the correlation coefficient of two PFAS, which is also indicated by the shade of each oval (blue are positive, red are negative), and the slant of the oval (pointing to the right upper corner is positive; pointing to the right lower corner is negative. Only highly detected PFAS were included. Number of stars represent the uncorrected p-value. * denotes <0.05; ** denotes <0.01; *** denotes <0.001. Abbreviations: PFAS – per-/polyfluoroalkyl substances; PFHxS – perfluorohexanesulphonic acid; PFDA – perfluorodecanoic acid; PFNA – perfluorononanoic acid; PFOA – perfluorooctanoic acid; PFOS – perfluorooctanesulfonic acid.

**Table S3: Genomic Inflation Factors (Lambdas) for Total Methylation Analysis**

| PFAS | Lambdas |
| --- | --- |
| PFHxS | 0.929 |
| PFOS | 1.034 |
| PFOA | 0.905 |
| PFNA | 1.236 |
| PFDA | 0.989 |
| PFUnDA | 0.987 |
| MeFOSAA | 1.050 |

*Observed p-values were tested for bias (inflation or deflation) by calculating the genomic inflation factor using results across all CpG sites with each PFAS (n=141 participants). Abbreviations: MeFOSAA – 2-(N-methyl-perfluorooctane sulfonamido) acetic acid; PFAS – per-/polyfluoroalkyl substances; PFHxS – perfluorohexanesulphonic acid; PFDA – perfluorodecanoic acid; PFNA – perfluorononanoic acid; PFOA – perfluorooctanoic acid; PFOS – perfluorooctanesulfonic acid; PFUnDA – perfluoroundecanoic acid.*

**Fig. S4: Comparison of estimates relating total methylation to individual per-/polyfluoroalkyl substances (PFAS) from the present birth study (main analysis, n=141) and previously published work.** A) Compares the coefficient estimates from individual CpG sites that were previously reported to be significantly related to individual PFAS to the estimates from the current study. Each shape denotes a different study. Details of comparison are located in Excel Table S1. B) Details the number of coefficients that were reported to be decreased in both previously reported literature and the current study (blue); increased in both previously reported literature and the current study (red); and reported to have opposite directions in previously reported literature and the current study (grey).


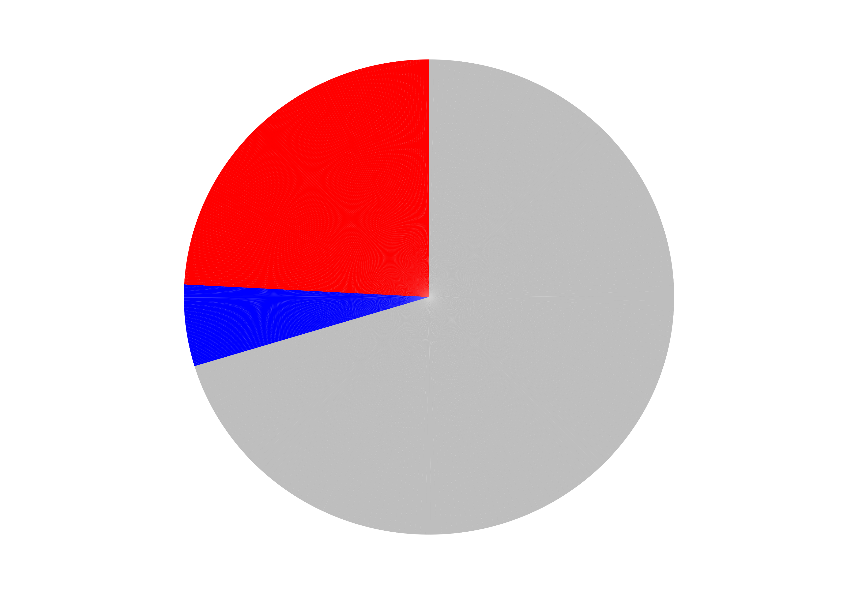

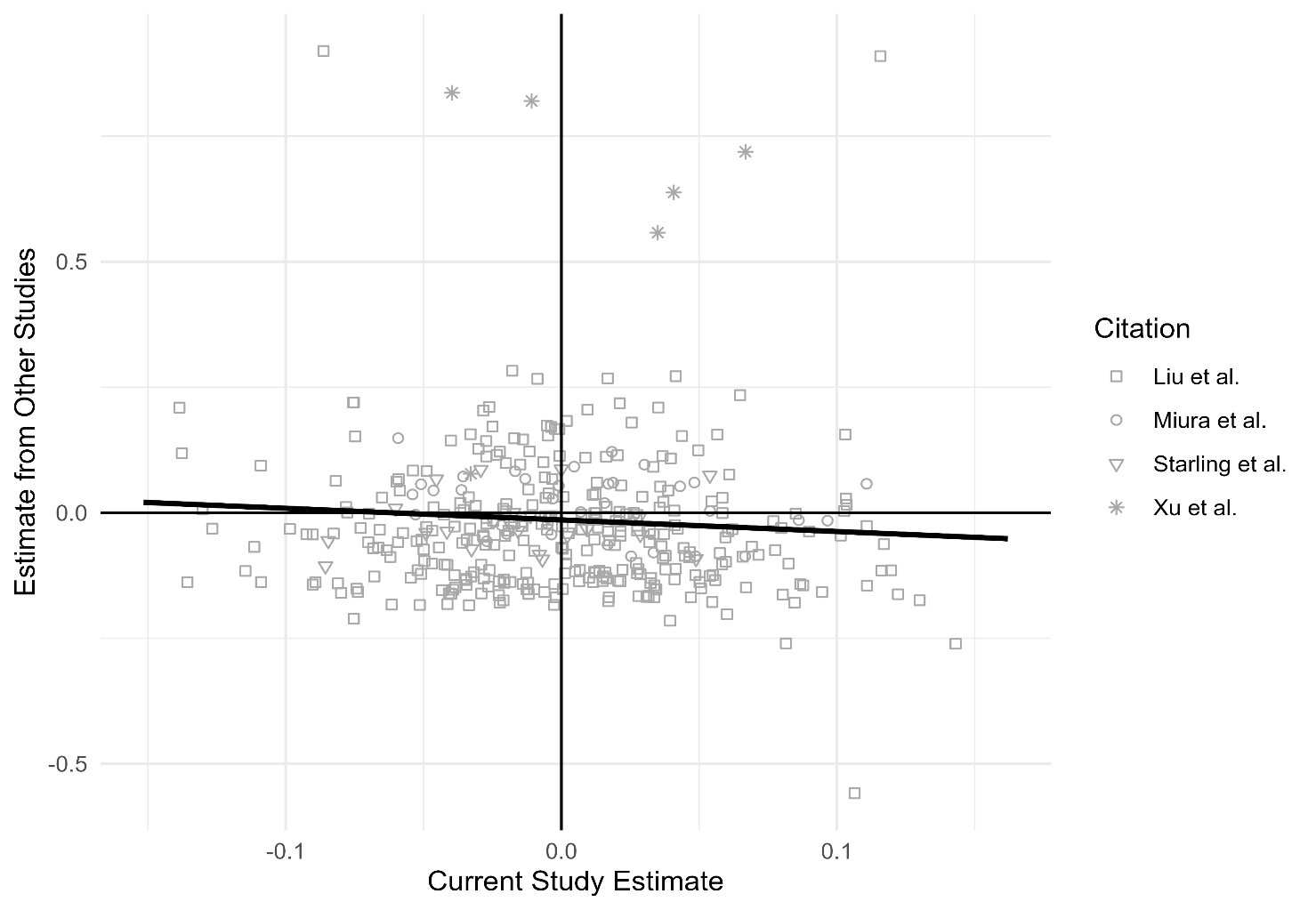


Opposite Directions

53.5% (203)

Both Decreased

31.4% (119)

Both Increased

15.0% (57)

**A**

**B**


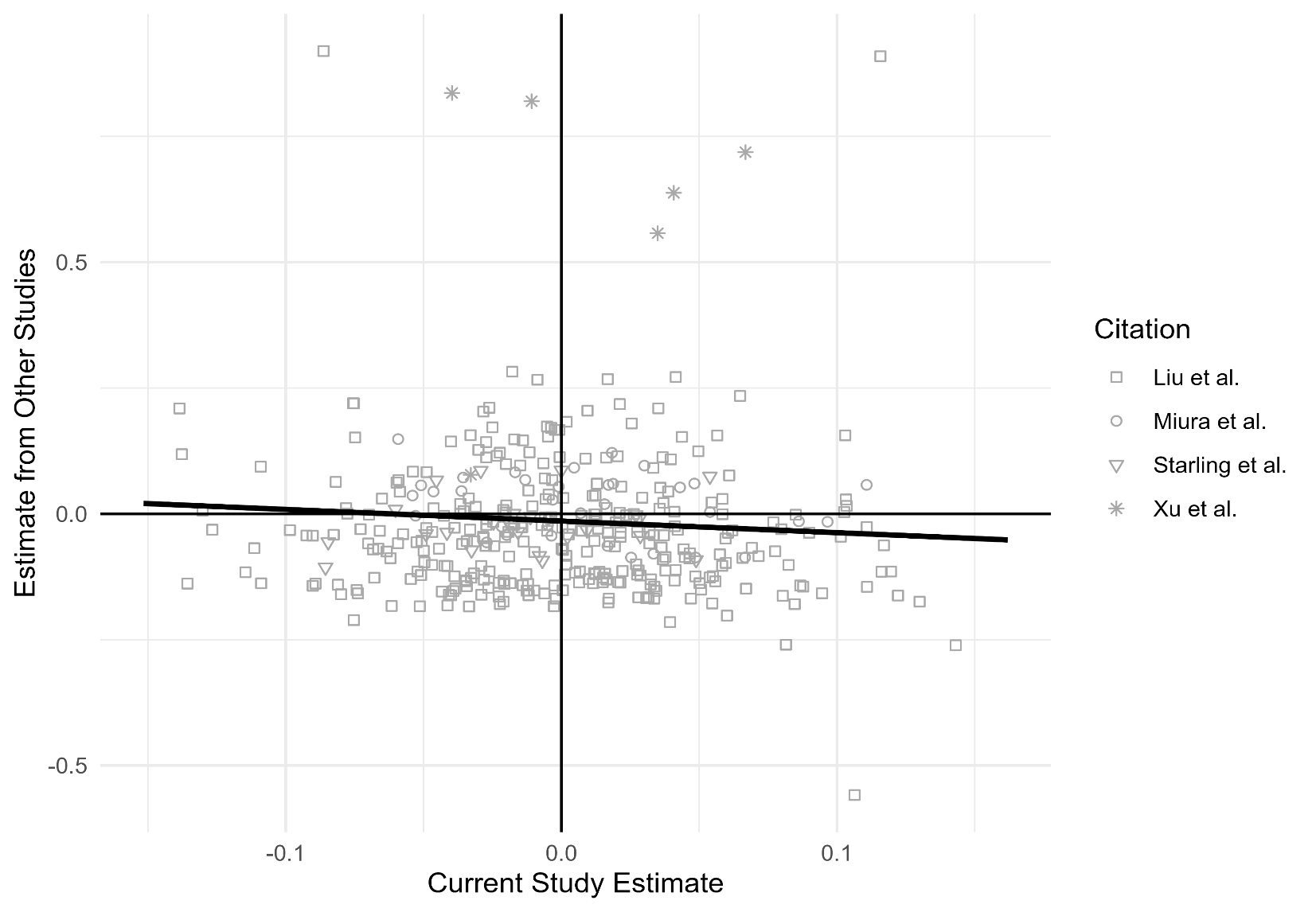


**Table S4: Sites with Significant Associations between PFAS and Total Methylation among females (*q*<0.05, n=72)**

| PFAS | Illumina CpG Name | Estimate | SE | t-value | p-value | BH q-value | Chromosome: Position | UCSC Gene Name | Relation to CpG Island* | Relation to Gene |
| --- | --- | --- | --- | --- | --- | --- | --- | --- | --- | --- |
| PFHxS |  |  |  |  |  |  |  |  |  |  |
|  | cg16940259 | -0.1566 | 0.0480 | -3.2627 | 0.0018 | 0.0117 | chr1:236323313 | *GPR137B* | OpenSea | 5'UTR |
|  | cg11973877 | -0.1514 | 0.0509 | -2.9744 | 0.0042 | 0.0252 | chr1:52195423 | *OSBPL9* | Island | TSS1500 |
|  | cg00119557 | -0.2334 | 0.0654 | -3.5692 | 0.0007 | 0.0059 | chr1:201751714 | *RNU6-79P; NAV1* | OpenSea | TSS200 |
|  | cg12570942 | 0.1937 | 0.0661 | 2.9302 | 0.0048 | 0.0267 | chr2:242626270 | *DTYMK* | Island | TSS1500 |
|  | cg13716787 | -0.2324 | 0.0853 | -2.7238 | 0.0084 | 0.0415 | chr2:70351227 | *LOC100133985* | N_Shore | 3'UTR |
|  | cg05904194 | 0.1939 | 0.0580 | 3.3421 | 0.0014 | 0.0100 | chr2:85132663 | *TMSB10* | Island | TSS200 |
|  | cg27350042 | 0.1758 | 0.0501 | 3.5127 | 0.0008 | 0.0064 | chr4:140033905 | *ELF2* | N_Shelf | 3'UTR |
|  | cg06706183 | 0.2341 | 0.0614 | 3.8153 | 0.0003 | 0.0038 | chr6:53409856 | *GCLC* | Island | 1stExon |
|  | cg11790979 | -0.3468 | 0.0661 | -5.2448 | 2.07E-06 | 0.0002 | chr7:150076338 | *ZNF775* | Island | TSS200 |
|  | cg09042952 | -0.2748 | 0.0660 | -4.1618 | 0.0001 | 0.0028 | chr12:122237463 | *LOC338799* | N_Shore | 3'UTR |
|  | cg19115132 | 0.3320 | 0.0654 | 5.0798 | 3.83E-06 | 0.0002 | chr15:45879518 | *BLOC1S6* | Island | TSS1500 |
|  | cg01603912 | 0.2030 | 0.0549 | 3.6999 | 0.0005 | 0.0049 | chr16:88039185 | *BANP* | N_Shore | 3'UTR |
|  | cg19231170 | -0.2271 | 0.0592 | -3.8371 | 0.0003 | 0.0038 | chr17:77020037 | *C1QTNF1* | Island | TSS1500 |
|  | cg02699671 | -0.1854 | 0.0515 | -3.6032 | 0.0006 | 0.0059 | chr17:850251 | *NXN* | Island | 3'UTR |
|  | cg08054038 | 0.2035 | 0.0514 | 3.9606 | 0.0002 | 0.0038 | chr19:45348674 | *PVRL2* | N_Shore | TSS1500 |
|  | cg03354590 | 0.2117 | 0.0542 | 3.9027 | 0.0002 | 0.0038 | chr19:46932167 |  | Island |  |
|  | cg09728659 | 0.1263 | 0.0450 | 2.8086 | 0.0067 | 0.0350 | chr22:18924873 | *PRODH* | S_Shore | TSS1500 |
| PFOS |  |  |  |  |  |  |  |  |  |  |
|  | cg10282673 | 0.1043 | 0.0422 | 2.4706 | 0.0163 | 0.0205 | chr1:65612682 | *AK4* | N_Shore | TSS1500 |
|  | cg24038158 | -0.6930 | 0.0995 | -6.9650 | 2.66E-09 | 6.52E-08 | chr1:23696199 | *C1orf213* | S_Shore | TSS1500 |
|  | cg11153969 | 0.4277 | 0.0687 | 6.2287 | 4.81E-08 | 3.62E-07 | chr1:207277031 | *C4BPA* | OpenSea | TSS1500 |
|  | cg24229231 | 0.6139 | 0.0911 | 6.7393 | 6.49E-09 | 9.36E-08 | chr1:236723358 | *HEATR1* | OpenSea |  |
|  | cg13821733 | 0.3628 | 0.0703 | 5.1575 | 2.87E-06 | 4.68E-06 | chr1:160314350 | *NCSTN; COPA* | S_Shore | 5'UTR |
|  | cg12909455 | 0.8726 | 0.1270 | 6.8710 | 3.86E-09 | 7.56E-08 | chr1:32458635 |  | OpenSea |  |
|  | cg26546906 | 0.3720 | 0.0720 | 5.1685 | 2.76E-06 | 4.58E-06 | chr1:229997149 |  | OpenSea |  |
|  | cg03244210 | 0.5887 | 0.0948 | 6.2098 | 5.18E-08 | 3.62E-07 | chr2:74035104 | *C2orf78* | OpenSea | TSS1500 |
|  | cg09929408 | 0.5736 | 0.0933 | 6.1466 | 6.62E-08 | 4.05E-07 | chr2:74035110 | *C2orf78* | OpenSea | TSS1500 |
|  | cg15965634 | 0.2707 | 0.0557 | 4.8609 | 8.54E-06 | 1.29E-05 | chr2:201992999 | *CFLAR* | OpenSea | 5'UTR |
|  | cg25545699 | 0.3788 | 0.0628 | 6.0363 | 1.02E-07 | 4.74E-07 | chr2:161132348 | *RBMS1* | OpenSea | 3'UTR |
|  | cg04980991 | -0.1974 | 0.0430 | -4.5884 | 2.28E-05 | 3.10E-05 | chr2:220035759 | *SLC23A3* | OpenSea | TSS1500 |
|  | cg03388879 | 0.4263 | 0.0702 | 6.0698 | 8.92E-08 | 4.61E-07 | chr2:201175772 | *SPATS2L* | S_Shelf | 5'UTR |
|  | cg05368609 | 0.3740 | 0.0651 | 5.7441 | 3.13E-07 | 9.59E-07 | chr2:201236639 | *SPATS2L* | OpenSea | TSS1500 |
|  | cg12079241 | 0.4525 | 0.0795 | 5.6936 | 3.80E-07 | 1.10E-06 | chr2:223230064 |  | OpenSea |  |
|  | cg02377589 | 0.2887 | 0.0693 | 4.1688 | 9.83E-05 | 0.0001 | chr2:2734065 |  | OpenSea | 3'UTR |
|  | cg10141352 | 0.3678 | 0.0649 | 5.6699 | 4.16E-07 | 1.10E-06 | chr3:155859344 | *KCNAB1* | OpenSea | TSS1500 |
|  | cg04808896 | 0.3481 | 0.0657 | 5.2959 | 1.71E-06 | 3.05E-06 | chr3:40141475 | *MYRIP* | OpenSea | TSS200 |
|  | cg07126979 | 0.3033 | 0.0660 | 4.5931 | 2.24E-05 | 3.09E-05 | chr3:127358879 | *PODXL2* | OpenSea |  |
|  | cg22714078 | 0.5702 | 0.0918 | 6.2140 | 5.09E-08 | 3.62E-07 | chr4:24540791 | *DHX15* | OpenSea | TSS200 |
|  | cg15838301 | 0.4291 | 0.0741 | 5.7902 | 2.62E-07 | 8.86E-07 | chr4:7719899 | *SORCS2* | OpenSea | ExonBnd |
|  | cg24727269 | 0.3876 | 0.0707 | 5.4798 | 8.57E-07 | 1.91E-06 | chr5:132588630 | *FSTL4* | OpenSea | TSS1500 |
|  | cg21685054 | 0.6873 | 0.1150 | 5.9782 | 1.27E-07 | 5.66E-07 | chr5:169810494 | *KCNMB1* | OpenSea | 3'UTR |
|  | cg21620108 | 0.5506 | 0.1068 | 5.1536 | 2.91E-06 | 4.68E-06 | chr5:1462734 | *LPCAT1* | N_Shore | 3'UTR |
|  | cg26157972 | -1.3030 | 0.1712 | -7.6108 | 2.06E-10 | 1.84E-08 | chr5:1049232 |  | OpenSea |  |
|  | cg26467752 | 0.2074 | 0.0422 | 4.9088 | 7.17E-06 | 1.10E-05 | chr5:2530020 |  | OpenSea |  |
|  | cg18281708 | 0.3232 | 0.0689 | 4.6920 | 1.57E-05 | 2.23E-05 | chr5:123286596 |  | OpenSea |  |
|  | cg13535098 | -0.7650 | 0.1026 | -7.4589 | 3.76E-10 | 1.84E-08 | chr6:3000053 | *NQO2* | N_Shore | TSS1500 |
|  | cg07553518 | 0.4092 | 0.0722 | 5.6702 | 4.15E-07 | 1.10E-06 | chr6:90628418 |  | OpenSea | TSS1500 |
|  | cg01630266 | 0.3181 | 0.0638 | 4.9820 | 5.49E-06 | 8.54E-06 | chr6:128260339 |  | OpenSea | 5'UTR |
|  | cg09031390 | 0.4691 | 0.0760 | 6.1743 | 5.94E-08 | 3.88E-07 | chr7:101861508 | *CUX1* | OpenSea |  |
|  | cg06002665 | 0.4593 | 0.0846 | 5.4315 | 1.03E-06 | 2.10E-06 | chr7:43352591 | *HECW1* | OpenSea | 5'UTR |
|  | cg13030011 | 0.4630 | 0.0763 | 6.0690 | 8.95E-08 | 4.61E-07 | chr7:23450804 | *IGF2BP3* | N_Shelf | 3'UTR |
|  | cg22064390 | 0.3977 | 0.0688 | 5.7773 | 2.76E-07 | 8.86E-07 | chr7:2615547 | *IQCE* | OpenSea | 5'UTR |
|  | cg19920283 | -0.6426 | 0.0917 | -7.0042 | 2.28E-09 | 6.52E-08 | chr7:105172520 | *RINT1* | Island | TSS200 |
|  | cg12892914 | 0.3823 | 0.0703 | 5.4360 | 1.01E-06 | 2.10E-06 | chr7:75484562 |  | OpenSea | 5'UTR |
|  | cg25591742 | 0.3880 | 0.0636 | 6.0975 | 8.01E-08 | 4.61E-07 | chr8:67405259 | *C8orf46* | OpenSea | TSS1500 |
|  | cg13473157 | 0.3664 | 0.0754 | 4.8573 | 8.65E-06 | 1.29E-05 | chr8:12805209 | *C8orf79* | N_Shelf | 5'UTR |
|  | cg05747874 | 0.4557 | 0.0804 | 5.6708 | 4.15E-07 | 1.10E-06 | chr8:22677410 | *PEBP4* | OpenSea | 3'UTR |
|  | cg07107633 | 0.5273 | 0.0785 | 6.7182 | 7.05E-09 | 9.36E-08 | chr8:40960365 |  | OpenSea |  |
|  | cg08475401 | 0.5176 | 0.1009 | 5.1308 | 3.17E-06 | 5.01E-06 | chr8:2143741 |  | OpenSea |  |
|  | cg09993605 | 0.4074 | 0.0674 | 6.0438 | 9.86E-08 | 4.74E-07 | chr9:7593062 |  | OpenSea |  |
|  | cg09730912 | 0.6157 | 0.1151 | 5.3475 | 1.41E-06 | 2.66E-06 | chr9:16131302 |  | OpenSea |  |
|  | cg06354984 | 0.7810 | 0.1338 | 5.8380 | 2.18E-07 | 7.93E-07 | chr10:128211107 | *C10orf90* | OpenSea | TSS1500 |
|  | cg04070599 | 0.3644 | 0.0788 | 4.6222 | 2.02E-05 | 2.83E-05 | chr10:13656608 | *PRPF18* | OpenSea | 5'UTR |
|  | cg14286486 | 0.3876 | 0.0656 | 5.9124 | 1.64E-07 | 6.69E-07 | chr10:105125108 |  | N_Shelf |  |
|  | cg06931591 | 0.5820 | 0.1044 | 5.5720 | 6.04E-07 | 1.52E-06 | chr10:118980094 |  | S_Shelf |  |
|  | cg26502489 | 0.3831 | 0.0664 | 5.7729 | 2.80E-07 | 8.86E-07 | chr11:118048546 | *SCN2B* | OpenSea | TSS1500 |
|  | cg09238106 | 0.4925 | 0.0771 | 6.3848 | 2.61E-08 | 2.33E-07 | chr11:64242796 |  | OpenSea |  |
|  | cg25554719 | 0.3650 | 0.0675 | 5.4042 | 1.14E-06 | 2.24E-06 | chr11:8699492 |  | OpenSea | TSS1500 |
|  | cg09839279 | 0.3928 | 0.0720 | 5.4584 | 9.29E-07 | 1.98E-06 | chr12:125627357 | *AACS* | OpenSea | 3'UTR |
|  | cg01745755 | 0.5494 | 0.0996 | 5.5171 | 7.44E-07 | 1.78E-06 | chr12:123834083 | *SBNO1* | OpenSea | TSS1500 |
|  | cg26105128 | 0.3527 | 0.0598 | 5.8956 | 1.75E-07 | 6.86E-07 | chr12:132221229 | *SFRS8* | S_Shore | 5'UTR |
|  | cg09793269 | 0.6720 | 0.1190 | 5.6485 | 4.51E-07 | 1.16E-06 | chr12:105348269 |  | N_Shelf | 5'UTR |
|  | cg01458683 | 0.1685 | 0.0617 | 2.7323 | 0.0082 | 0.0107 | chr13:76112187 | *COMMD6* | S_Shore | 5'UTR |
|  | cg18772379 | 0.4061 | 0.0741 | 5.4800 | 8.56E-07 | 1.91E-06 | chr13:51480138 |  | N_Shelf | 3'UTR |
|  | cg17286831 | 0.4996 | 0.0751 | 6.6534 | 9.10E-09 | 9.91E-08 | chr14:65273399 | *SPTB* | OpenSea | 5'UTR |
|  | cg08764872 | -0.2645 | 0.0496 | -5.3374 | 1.47E-06 | 2.71E-06 | chr14:22446527 |  | OpenSea | TSS200 |
|  | cg01362149 | 0.4330 | 0.0902 | 4.7998 | 1.07E-05 | 1.54E-05 | chr14:105550889 |  | N_Shelf |  |
|  | cg02498211 | 0.0933 | 0.0246 | 3.7996 | 0.0003 | 0.0004 | chr14:55665444 |  | OpenSea | 3'UTR |
|  | cg25250717 | 0.3206 | 0.0593 | 5.4080 | 1.12E-06 | 2.24E-06 | chr15:61144420 | *RORA* | OpenSea | 3'UTR |
|  | cg10182135 | 0.4542 | 0.0762 | 5.9627 | 1.35E-07 | 5.75E-07 | chr16:68725578 | *CDH3* | OpenSea | TSS200 |
|  | cg11280185 | 0.6982 | 0.1042 | 6.6978 | 7.64E-09 | 9.36E-08 | chr16:237270 |  | Island |  |
|  | cg18996153 | 0.3697 | 0.0712 | 5.1900 | 2.54E-06 | 4.37E-06 | chr17:80144253 | *CCDC57* | OpenSea | TSS1500 |
|  | cg21864868 | 0.3505 | 0.0638 | 5.4917 | 8.19E-07 | 1.91E-06 | chr17:46673002 | *LOC404266* | N_Shore | 5'UTR |
|  | cg07905199 | 0.4602 | 0.0809 | 5.6892 | 3.86E-07 | 1.10E-06 | chr17:67507798 | *MAP2K6* | OpenSea |  |
|  | cg14123409 | 0.3192 | 0.0546 | 5.8501 | 2.08E-07 | 7.86E-07 | chr17:2831903 | *RAP1GAP2* | OpenSea |  |
|  | cg21668653 | 0.5710 | 0.1044 | 5.4686 | 8.94E-07 | 1.95E-06 | chr17:2773306 | *RAP1GAP2* | OpenSea |  |
|  | cg09057359 | 0.3978 | 0.0720 | 5.5242 | 7.24E-07 | 1.77E-06 | chr17:53032434 | *TOM1L1* | OpenSea | 1stExon |
|  | cg02196189 | 0.4037 | 0.0622 | 6.4925 | 1.71E-08 | 1.68E-07 | chr18:13121061 | *CEP192* | OpenSea | 5'UTR |
|  | cg00416475 | 0.3855 | 0.0744 | 5.1783 | 2.66E-06 | 4.49E-06 | chr19:19745573 | *GMIP* | Island |  |
|  | cg13701180 | 0.3810 | 0.0791 | 4.8183 | 9.97E-06 | 1.46E-05 | chr19:2513436 | *GNG7* | Island | 3'UTR |
|  | cg05278271 | -0.1194 | 0.0472 | -2.5282 | 0.0141 | 0.0179 | chr19:7554137 | *PEX11G* | S_Shore | TSS1500 |
|  | cg02400474 | 0.0957 | 0.0356 | 2.6909 | 0.0092 | 0.0118 | chr19:58842213 | *ZSCAN22* | S_Shelf | 5'UTR |
|  | cg24423230 | 0.3608 | 0.0672 | 5.3724 | 1.29E-06 | 2.47E-06 | chr20:50794730 | *ZFP64* | OpenSea | 3'UTR |
|  | cg25644372 | 0.3809 | 0.0732 | 5.2038 | 2.42E-06 | 4.23E-06 | chr20:1514061 |  | OpenSea |  |
|  | cg26276791 | 0.3255 | 0.0611 | 5.3259 | 1.53E-06 | 2.78E-06 | chr22:29388204 | *ZNRF3* | OpenSea | TSS1500 |
|  | cg01128923 | 0.4399 | 0.0756 | 5.8155 | 2.38E-07 | 8.33E-07 | chr22:46289831 |  | S_Shelf |  |
| PFNA |  |  |  |  |  |  |  |  |  |  |
|  | cg09057359 | 0.4517 | 0.0729 | 6.1981 | 5.42E-08 | 2.71E-07 | chr17:53032434 | *TOM1L1; COX11* | OpenSea | 1stExon |
| PFDA |  |  |  |  |  |  |  |  |  |  |
|  | cg25260255 | -0.2951 | 0.0600 | -4.9145 | 7.02E-06 | 3.51E-05 | chr6:31670897 | *BAT5* | Island | TSS200 |
|  | cg19547236 | -0.1545 | 0.0344 | -4.4940 | 3.18E-05 | 7.95E-05 | chr19:19175039 | *SLC25A42* | S_Shore | 5'UTR |
| PFUnDA |  |  |  |  |  |  |  |  |  |  |
|  | cg14134364 | -0.0745 | 0.0338 | -2.2008 | 0.0315 | 0.0315 | chr1:248110997 | *OR2L8* | OpenSea | TSS1500 |
|  | cg26557737 | -0.1998 | 0.0443 | -4.5134 | 2.97E-05 | 5.94E-05 | chr1:158463096 |  | OpenSea | 3'UTR |

**Annotations relative to CpG Islands: OpenSea= not within an island region; Island = within a CpG island; N_Shore or S_Shore = North or South Shore upstream/downstream of CpG Island; N_Shelf or S_Shelf= North or South Shelf upstream/downstream of CpG island.*

*Abbreviations: BH – Benjamini-Hotchberg; CpG – cytosine-guanine site of methylation; ExonBnd – exon boundary; PFAS – per-/polyfluoroalkyl substances; PFHxS – perfluorohexanesulphonic acid; PFDA – perfluorodecanoic acid; PFNA – perfluorononanoic acid; PFOS – perfluorooctanesulfonic acid; SE – standard error; TSS – transcription start site; UTR – untranslated region.*

**Table S5: Sites with Significant Associations between PFAS and Total Methylation among Males (*q*<0.05, n=69)**

| PFAS | Illumina CpG Name | Estimate | SE | t-value | p-value | BH q-value | Chromosome: Position | UCSC Gene Name | Relation to CpG Island* | Relation to Gene |
| --- | --- | --- | --- | --- | --- | --- | --- | --- | --- | --- |
| PFHxS |  |  |  |  |  |  |  |  |  |  |
|  | cg16589617 | -0.5324 | 0.0774 | -6.8815 | 4.63E-09 | 9.98E-09 | chr1:34339784 | *CSMD2-AS1* | OpenSea | 5'UTR |
|  | cg20785560 | -0.1326 | 0.0647 | -2.0497 | 0.045 | 0.047 | chr1:78511235 | *GIPC2* | N_Shore | TSS1500 |
|  | cg16940259 | 0.1802 | 0.0537 | 3.3527 | 0.001 | 0.002 | chr1:236323313 | *GPR137B* | OpenSea | 5'UTR |
|  | cg26616083 | 0.1974 | 0.0468 | 4.2210 | 8.67E-05 | 0.0001 | chr1:26736154 | *LIN28* | N_Shore | TSS1500 |
|  | cg11973877 | 0.1989 | 0.0582 | 3.4195 | 0.001 | 0.001 | chr1:52195423 | *OSBPL9* | Island | TSS1500 |
|  | cg23629959 | -0.5302 | 0.0906 | -5.8543 | 2.37E-07 | 3.90E-07 | chr1:84644512 | *PRKACB* | OpenSea | TSS200 |
|  | cg14253939 | -0.3663 | 0.0677 | -5.4081 | 1.26E-06 | 1.99E-06 | chr1:228674594 | *RNF187* | Island | TSS1500 |
|  | cg00119557 | 0.1677 | 0.0509 | 3.2919 | 0.002 | 0.002 | chr1:201751714 | *RNU6-79P; NAV1* | OpenSea | TSS200 |
|  | cg09799337 | -1.0940 | 0.1466 | -7.4633 | 4.88E-10 | 1.46E-09 | chr1:155978343 |  | OpenSea | TSS1500 |
|  | cg09486778 | 1.1179 | 0.1287 | 8.6860 | 4.39E-12 | 4.10E-11 | chr2:223169609 | *CCDC140* | N_Shore | 3'UTR |
|  | cg12570942 | -0.3126 | 0.0791 | -3.9549 | 0.0002 | 0.0003 | chr2:242626270 | *DTYMK* | Island | TSS1500 |
|  | cg01206211 | -0.5971 | 0.0876 | -6.8177 | 5.93E-09 | 1.21E-08 | chr2:36825736 | *FEZ2* | S_Shore | TSS1500 |
|  | cg05645702 | -1.1729 | 0.1394 | -8.4146 | 1.24E-11 | 8.71E-11 | chr2:242190905 | *HDLBP* | OpenSea | TSS1500 |
|  | cg13716787 | 0.3939 | 0.0802 | 4.9132 | 7.69E-06 | 1.17E-05 | chr2:70351227 | *LOC100133985* | N_Shore | 3'UTR |
|  | cg05904194 | -0.1860 | 0.0665 | -2.7984 | 0.007 | 0.008 | chr2:85132663 | *TMSB10* | Island | TSS200 |
|  | cg00858624 | -0.5014 | 0.0781 | -6.4201 | 2.74E-08 | 4.90E-08 | chr2:131608667 |  | OpenSea | 5'UTR |
|  | cg26143045 | -0.3538 | 0.0732 | -4.8305 | 1.03E-05 | 1.52E-05 | chr3:132757610 | *TMEM108* | Island | 5'UTR |
|  | cg27350042 | -0.1122 | 0.0370 | -3.0295 | 0.004 | 0.004 | chr4:140033905 | *ELF2* | N_Shelf | 3'UTR |
|  | cg17822633 | -0.2684 | 0.0620 | -4.3276 | 6.03E-05 | 8.30E-05 | chr4:151464300 | *LRBA* | OpenSea | 3'UTR |
|  | cg03282686 | 0.9074 | 0.0823 | 11.0205 | 7.49E-16 | 6.29E-14 | chr4:177116826 | *SPATA4* | Island | TSS1500 |
|  | cg22958231 | 0.6654 | 0.0900 | 7.3935 | 6.39E-10 | 1.68E-09 | chr4:177116835 | *SPATA4* | Island | TSS1500 |
|  | cg16451306 | -0.6709 | 0.0861 | -7.7920 | 1.37E-10 | 6.01E-10 | chr5:71475356 | *MAP1B* | OpenSea | TSS200 |
|  | cg21555240 | -1.1237 | 0.1336 | -8.4140 | 1.24E-11 | 8.71E-11 | chr5:89734950 |  | OpenSea |  |
|  | cg27370696 | 0.8105 | 0.0869 | 9.3233 | 3.90E-13 | 8.19E-12 | chr6:32146529 | *AGPAT1; RNF5* | OpenSea | TSS1500 |
|  | cg07482220 | 0.6775 | 0.0874 | 7.7509 | 1.60E-10 | 6.41E-10 | chr6:32146520 | *AGPAT1; RNF5* | OpenSea | TSS1500 |
|  | cg06570818 | 0.5927 | 0.0888 | 6.6773 | 1.02E-08 | 1.99E-08 | chr6:32146466 | *AGPAT1; RNF5* | OpenSea | TSS1500 |
|  | cg17455891 | 0.7112 | 0.0794 | 8.9572 | 1.56E-12 | 2.62E-11 | chr6:32146048 | *AGPAT1; RNF5P1* | OpenSea | TSS1500 |
|  | cg23464264 | 0.6006 | 0.0722 | 8.3217 | 1.77E-11 | 1.06E-10 | chr6:32145923 | *AGPAT1; RNF5P1* | OpenSea | TSS1500 |
|  | cg08049198 | 0.5429 | 0.0762 | 7.1258 | 1.80E-09 | 4.59E-09 | chr6:32145904 | *AGPAT1; RNF5P1* | OpenSea | TSS1500 |
|  | cg18928683 | 0.5749 | 0.0852 | 6.7483 | 7.75E-09 | 1.55E-08 | chr6:32146006 | *AGPAT1; RNF5P1* | OpenSea | TSS1500 |
|  | cg06706183 | -0.1980 | 0.0758 | -2.6116 | 0.011 | 0.012 | chr6:53409856 | *GCLC* | Island | 1stExon |
|  | cg06015579 | -0.2532 | 0.0410 | -6.1682 | 7.18E-08 | 1.23E-07 | chr6:2232775 | *GMDS* | OpenSea |  |
|  | cg24425483 | 0.5044 | 0.0721 | 6.9941 | 3.00E-09 | 7.00E-09 | chr6:32146195 | *RNF5; AGPAT1* | OpenSea | TSS1500 |
|  | cg13763617 | 1.0614 | 0.1106 | 9.5956 | 1.40E-13 | 3.92E-12 | chr6:32145755 | *RNF5P1; RNF5* | OpenSea | TSS200 |
|  | cg01052103 | 0.7273 | 0.0831 | 8.7564 | 3.35E-12 | 3.52E-11 | chr6:32145383 | *RNF5P1; RNF5* | OpenSea | TSS1500 |
|  | cg02260340 | 0.8295 | 0.0965 | 8.5971 | 6.17E-12 | 5.18E-11 | chr6:32145753 | *RNF5P1; RNF5* | OpenSea | TSS200 |
|  | cg01074928 | 0.6329 | 0.0761 | 8.3219 | 1.77E-11 | 1.06E-10 | chr6:32145543 | *RNF5P1; RNF5* | OpenSea | 5'UTR |
|  | cg08450897 | 0.6757 | 0.0897 | 7.5346 | 3.70E-10 | 1.15E-09 | chr6:32145626 | *RNF5P1; RNF5* | OpenSea | TSS200 |
|  | cg09301199 | 0.6314 | 0.0907 | 6.9637 | 3.37E-09 | 7.66E-09 | chr6:32145654 | *RNF5P1; RNF5* | OpenSea | TSS200 |
|  | cg11172857 | -1.0723 | 0.1402 | -7.6474 | 2.39E-10 | 8.74E-10 | chr6:168956903 | *SMOC2* | Island |  |
|  | cg24132222 | -0.5660 | 0.0740 | -7.6537 | 2.34E-10 | 8.74E-10 | chr6:35406192 |  | OpenSea |  |
|  | cg10269127 | 0.2278 | 0.0481 | 4.7327 | 1.47E-05 | 2.09E-05 | chr7:138552716 | *KIAA1549* | Island | ExonBnd |
|  | cg11790979 | 0.2076 | 0.0767 | 2.7071 | 0.009 | 0.010 | chr7:150076338 | *ZNF775* | Island | TSS200 |
|  | cg03886307 | -0.6891 | 0.0927 | -7.4376 | 5.39E-10 | 1.56E-09 | chr7:21165124 |  | OpenSea |  |
|  | cg16653966 | -0.5196 | 0.0759 | -6.8492 | 5.25E-09 | 1.10E-08 | chr8:68069776 | *CSPP1* | OpenSea | TSS200 |
|  | cg25153092 | -0.5805 | 0.0770 | -7.5399 | 3.63E-10 | 1.15E-09 | chr8:130960409 | *FAM49B* | OpenSea | 5'UTR |
|  | cg22990430 | 0.6397 | 0.0927 | 6.8990 | 4.33E-09 | 9.57E-09 | chr8:56433632 | *XKR4* | OpenSea | TSS200 |
|  | cg21053301 | -0.3512 | 0.0582 | -6.0342 | 1.20E-07 | 2.01E-07 | chr8:8310138 |  | OpenSea |  |
|  | cg02124746 | -1.1717 | 0.1444 | -8.1118 | 3.98E-11 | 1.97E-10 | chr9:91615137 | *S1PR3* | N_Shore | 5'UTR |
|  | cg23962250 | 1.0743 | 0.1638 | 6.5576 | 1.62E-08 | 3.02E-08 | chr10:134755955 | *C10orf93* | Island | 3'UTR |
|  | cg21903920 | -0.9730 | 0.1277 | -7.6206 | 2.65E-10 | 8.92E-10 | chr10:124145541 | *PLEKHA1* | OpenSea | TSS1500 |
|  | cg23788374 | -0.7872 | 0.0983 | -8.0087 | 5.92E-11 | 2.76E-10 | chr10:114498704 | *VTI1A* | OpenSea | 5'UTR |
|  | cg00267172 | -0.8437 | 0.1105 | -7.6327 | 2.53E-10 | 8.86E-10 | chr10:11487791 |  | OpenSea |  |
|  | cg07343438 | -0.5519 | 0.0873 | -6.3220 | 3.99E-08 | 6.98E-08 | chr10:43815780 |  | N_Shelf |  |
|  | cg06818975 | -0.6351 | 0.0718 | -8.8479 | 2.37E-12 | 3.31E-11 | chr11:66063091 | *TMEM151A* | Island |  |
|  | cg06267084 | -0.6383 | 0.0964 | -6.6242 | 1.25E-08 | 2.39E-08 | chr11:392508 |  | N_Shore | TSS200 |
|  | cg12391352 | 0.7300 | 0.0987 | 7.3979 | 6.28E-10 | 1.68E-09 | chr12:99139768 | *ANKS1B* | Island | TSS200 |
|  | cg20725662 | -1.0459 | 0.1344 | -7.7803 | 1.43E-10 | 6.01E-10 | chr12:978764 | *HSN2* | OpenSea | 5'UTR |
|  | cg09042952 | 0.1516 | 0.0458 | 3.3090 | 0.002 | 0.002 | chr12:122237463 | *LOC338799* | N_Shore | 3'UTR |
|  | cg26214972 | -0.6460 | 0.1239 | -5.2142 | 2.57E-06 | 4.00E-06 | chr12:120780777 | *MSI1* | OpenSea | 3'UTR |
|  | cg04792963 | -0.8070 | 0.0921 | -8.7584 | 3.33E-12 | 3.52E-11 | chr12:133015383 |  | Island |  |
|  | cg01603912 | -0.2024 | 0.0582 | -3.4787 | 0.001 | 0.001 | chr16:88039185 | *BANP* | N_Shore | 3'UTR |
|  | cg04919263 | 0.7664 | 0.0938 | 8.1723 | 3.15E-11 | 1.65E-10 | chr16:3086292 | *CCDC64B* | Island | 5'UTR |
|  | cg16517195 | -0.4586 | 0.0710 | -6.4604 | 2.35E-08 | 4.29E-08 | chr16:66429972 | *CDH5* | OpenSea | 5'UTR |
|  | cg07493596 | 0.2671 | 0.0664 | 4.0237 | 0.0002 | 0.0002 | chr16:85722677 | *GINS2* | Island | TSS200 |
|  | cg26801741 | -0.4008 | 0.0729 | -5.4991 | 8.98E-07 | 1.45E-06 | chr16:12252833 | *SNX29* | OpenSea |  |
|  | cg03444587 | -0.5911 | 0.0841 | -7.0267 | 2.64E-09 | 6.34E-09 | chr16:2054271 | *ZNF598* | Island | 3'UTR |
|  | cg19231170 | 0.2377 | 0.0729 | 3.2612 | 0.002 | 0.002 | chr17:77020037 | *C1QTNF1* | Island | TSS1500 |
|  | cg12838546 | 0.1992 | 0.0412 | 4.8379 | 1.01E-05 | 1.51E-05 | chr17:40713978 | *COASY* | N_Shore | TSS200 |
|  | cg06554293 | -0.5776 | 0.0816 | -7.0749 | 2.19E-09 | 5.42E-09 | chr17:6531884 | *KIAA0753* | OpenSea | TSS1500 |
|  | cg02699671 | 0.1371 | 0.0440 | 3.1167 | 0.003 | 0.003 | chr17:850251 | *NXN* | Island | 3'UTR |
|  | cg13251490 | 0.2551 | 0.0645 | 3.9546 | 0.0002 | 0.0003 | chr17:1531887 | *SLC43A2* | Island | 5'UTR |
|  | cg20245757 | 0.3172 | 0.0702 | 4.5204 | 3.09E-05 | 4.33E-05 | chr17:46663759 |  | S_Shelf | 5'UTR |
|  | cg26853093 | 1.0800 | 0.1052 | 10.2710 | 1.14E-14 | 4.79E-13 | chr19:17448469 | *GTPBP3* | Island | 1stExon |
|  | cg05411953 | 0.1742 | 0.0470 | 3.7041 | 0.0005 | 0.0006 | chr19:52207653 | *NCRNA00085* | Island | 5'UTR |
|  | cg08054038 | -0.1860 | 0.0525 | -3.5466 | 0.0008 | 0.0010 | chr19:45348674 | *PVRL2* | N_Shore | TSS1500 |
|  | cg25979157 | -0.6102 | 0.0824 | -7.4093 | 6.01E-10 | 1.68E-09 | chr19:53902687 | *ZNF765* | S_Shelf | 5'UTR |
|  | cg02384543 | -0.8209 | 0.0989 | -8.3025 | 1.91E-11 | 1.07E-10 | chr19:58879857 | *ZNF837* | Island |  |
|  | cg03354590 | -0.1356 | 0.0580 | -2.3364 | 0.023 | 0.024 | chr19:46932167 |  | Island |  |
|  | cg21395191 | -0.3032 | 0.0639 | -4.7465 | 1.40E-05 | 2.02E-05 | chr22:50608218 | *PANX2* | Island | TSS1500 |
|  | cg09728659 | -0.1815 | 0.0444 | -4.0847 | 0.0001 | 0.0002 | chr22:18924873 | *PRODH* | S_Shore | TSS1500 |
| PFOS |  |  |  |  |  |  |  |  |  |  |
|  | cg04789125 | 0.5680 | 0.1510 | 3.7611 | 0.000 | 0.004 | chr2:242798512 | *PDCD1* | N_Shore |  |
|  | cg00075620 | 0.4166 | 0.1081 | 3.8535 | 0.000 | 0.004 | chr2:137180506 |  | N_Shore |  |
|  | cg04482003 | -0.8955 | 0.1559 | -5.7442 | 3.59E-07 | 1.76E-05 | chr4:108852521 | *CYP2U1* | Island | TSS1500 |
|  | cg15831743 | -0.6268 | 0.1460 | -4.2929 | 6.79E-05 | 0.0011 | chr5:122421924 |  | N_Shelf |  |
|  | cg12355459 | 0.5771 | 0.1413 | 4.0847 | 0.000 | 0.002 | chr8:53548594 | *RB1CC1* | OpenSea | TSS200 |
|  | cg02124746 | -1.0345 | 0.1351 | -7.6569 | 2.31E-10 | 2.26E-08 | chr9:91615137 | *S1PR3* | N_Shore | 5'UTR |
|  | cg00138025 | 0.6899 | 0.1510 | 4.5691 | 2.61E-05 | 0.0005 | chr12:120219052 | *CIT* | OpenSea | 3'UTR |
|  | cg01458683 | -0.7365 | 0.1515 | -4.8602 | 9.30E-06 | 0.0003 | chr13:76112187 | *COMMD6* | S_Shore | 5'UTR |
|  | cg10150605 | 0.5977 | 0.1276 | 4.6845 | 1.74E-05 | 0.0004 | chr18:55834475 | *NEDD4L* | OpenSea | 5'UTR |
|  | cg02400474 | -0.0945 | 0.0323 | -2.9245 | 0.005 | 0.048 | chr19:58842213 | *ZSCAN22* | S_Shelf | 5'UTR |
| PFDA |  |  |  |  |  |  |  |  |  |  |
|  | cg25260255 | 0.1661 | 0.0510 | 3.2572 | 0.002 | 0.005 | chr6:31670897 | *BAT5* | Island | TSS200 |
|  | cg19547236 | 0.1547 | 0.0351 | 4.4028 | 4.65E-05 | 0.0002 | chr19:19175039 | *SLC25A42* | S_Shore | 5'UTR |
| PFUnDA |  |  |  |  |  |  |  |  |  |  |
|  | cg14134364 | 0.1607 | 0.0337 | 4.7654 | 1.30E-05 | 2.61E-05 | chr1:248110997 | *OR2L8* | OpenSea | TSS1500 |
|  | cg26557737 | 0.2277 | 0.0501 | 4.5415 | 2.87E-05 | 2.87E-05 | chr1:158463096 |  | OpenSea | 3'UTR |

**Annotations relative to CpG Islands: OpenSea= not within an island region; Island = within a CpG island; N_Shore or S_Shore = North or South Shore upstream/downstream of CpG Island; N_Shelf or S_Shelf= North or South Shelf upstream/downstream of CpG island.*

*Abbreviations: BH – Benjamini-Hotchberg; CpG – cytosine-guanine site of methylation; ExonBnd – exon boundary; PFAS – per-/polyfluoroalkyl substances; PFHxS – perfluorohexanesulphonic acid; PFDA – perfluorodecanoic acid; PFNA – perfluorononanoic acid; PFOS – perfluorooctanesulfonic acid; SE – standard error; TSS – transcription start site; UTR – untranslated region.*

**Table S6: Genomic Inflation Factors (Lambdas) for PFAS – Methylation Type (5-hmC and 5-mC) Interaction Analysis**

| PFAS | Lambdas |
| --- | --- |
| PFHxS | 0.859 |
| PFOS | 0.956 |
| PFOA | 0.911 |
| PFNA | 0.991 |
| PFDA | 1.168 |
| PFUA | 0.993 |
| MeFOSAA | 0.924 |

*Observed p-values for the interaction between exposure and type of DNA methylation were tested for bias (inflation or deflation) by calculating the genomic inflation factor using results across all CpG sites. N=70. Abbreviations: 5-mC – 5-methylcytosine; 5-hmC – 5-hydroxymethylcytosine; MeFOSAA – 2-(N-methyl-perfluorooctane sulfonamido) acetic acid; PFAS – per-/polyfluoroalkyl substances; PFHxS – perfluorohexanesulphonic acid; PFDA – perfluorodecanoic acid; PFNA – perfluorononanoic acid; PFOA – perfluorooctanoic acid; PFOS – perfluorooctanesulfonic acid; PFUnDA – perfluoroundecanoic acid.*


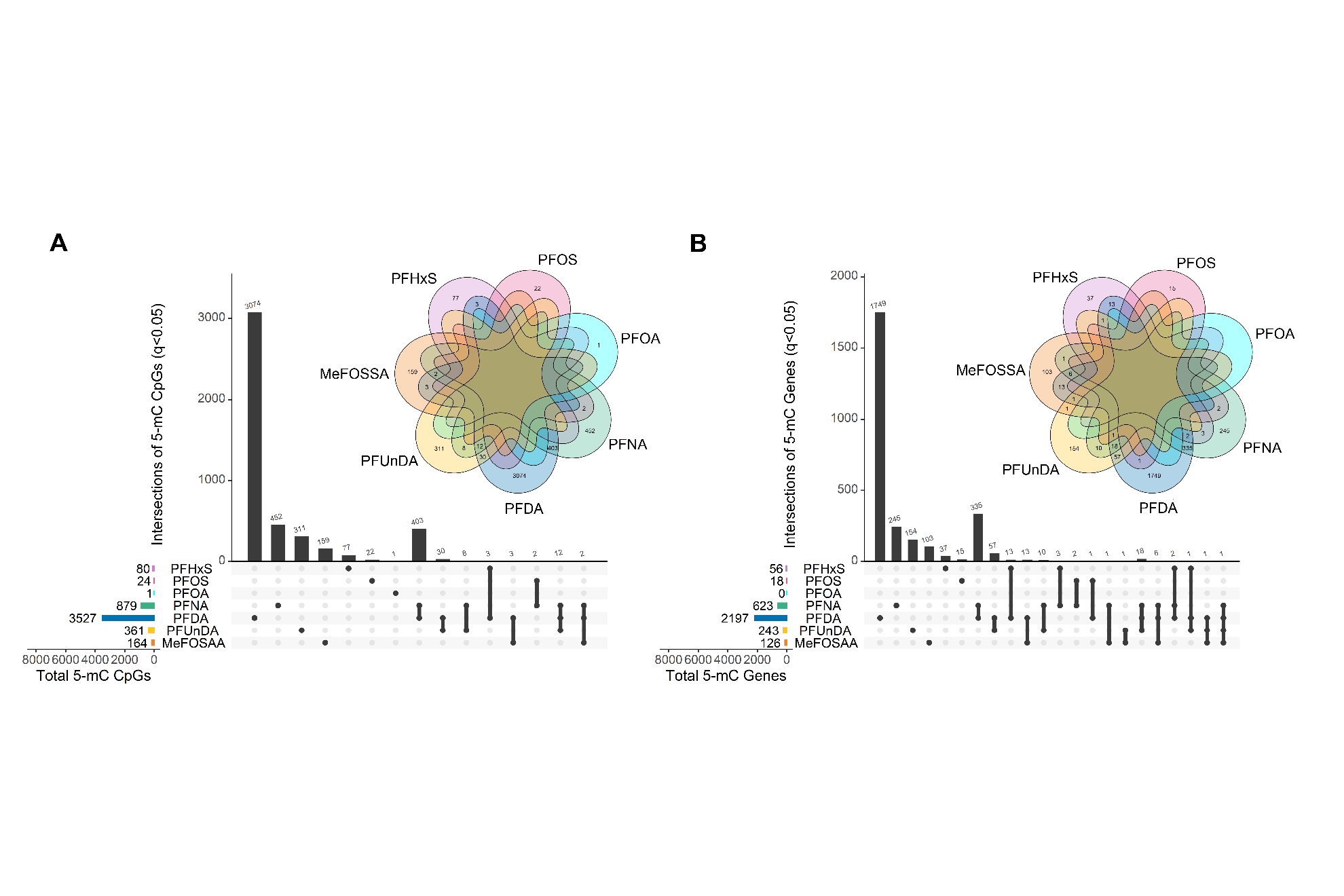


**Fig. S5: Overlap for significant 5-methylcytosine (5-mC, *q*<0.05) sites (A) and genes (B) by PFAS (n=70).** Each plot and Venn diagram inset shows the overlap between all PFAS in the present study. β corresponds the coefficient estimate, p represents the uncorrected p-value, and q represents the Benjamini-Hochberg corrected q-value. Abbreviations: MeFOSAA – 2-(N-methyl-perfluorooctane sulfonamido) acetic acid; PFAS– per-/polyfluoroalkyl substances; PFHxS – perfluorohexanesulphonic acid; PFDA – perfluorodecanoic acid; PFNA – perfluorononanoic acid; PFOS – perfluorooctanesulfonic acid; PFUnDA – perfluoroundecanoic acid.


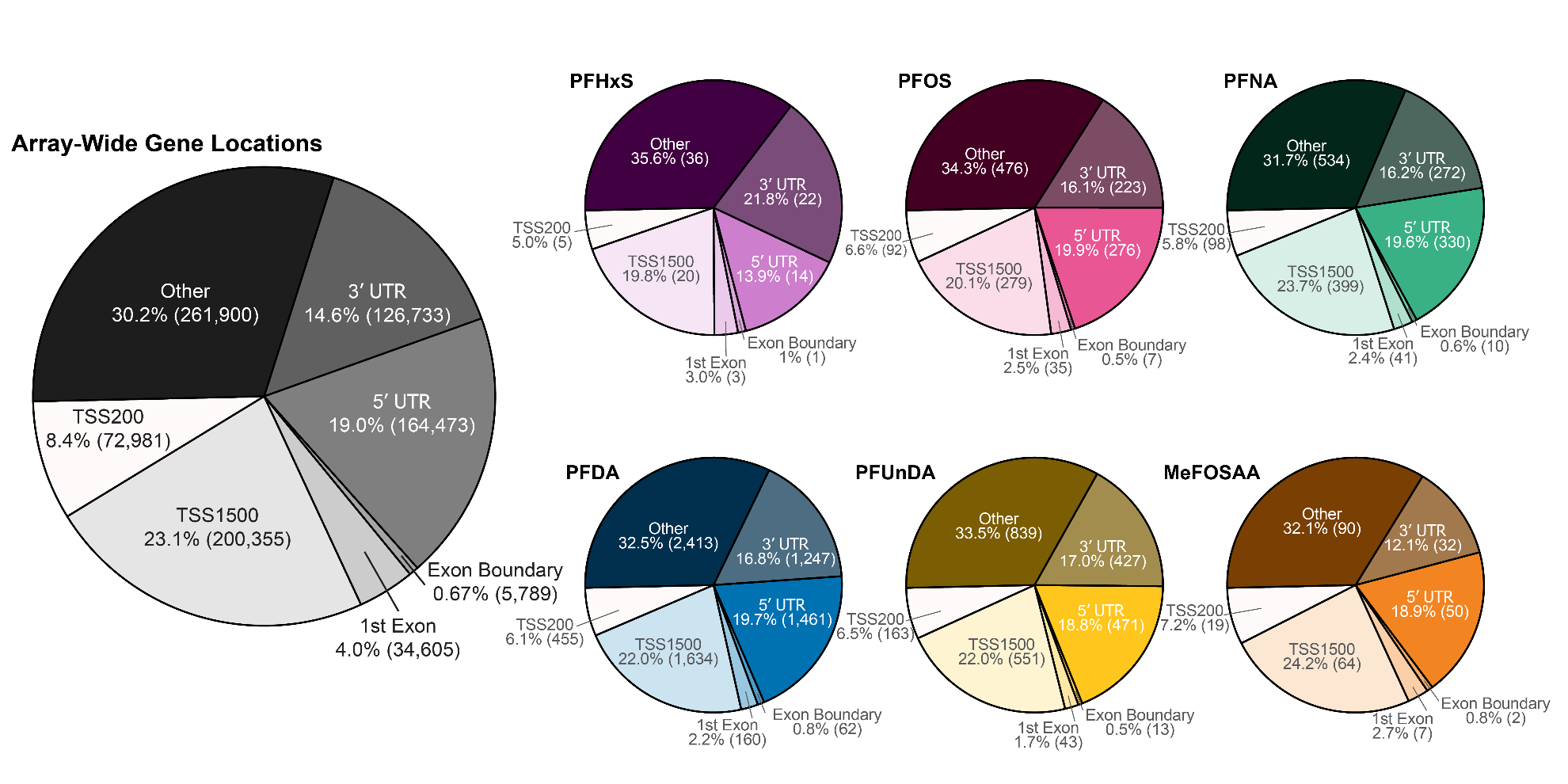


**Fig. S6: Gene locations for significant 5-hydroxymethylcytosine (5-hmC) sites by PFAS (*q*<0.05).** Each piechart shows location of significant sites in relation to genes) (n=70). Charts are labeled with percent of sites in each location and the total number of sites in each category in parentheses. TSS1500 (200–1500 bases upstream of the transcriptional start site), TSS200 (0-200 bases upstream of the transcriptional start site); 1st Exon (first exon of the gene); Exon Boundary; 5’UTR (untranslated region); 3’UTR; and other locations. Abbreviations: MeFOSAA – 2-(N-methyl-perfluorooctane sulfonamido) acetic acid; PFHxS – perfluorohexanesulphonic acid; PFDA – perfluorodecanoic acid; PFNA – perfluorononanoic acid; PFOS – perfluorooctanesulfonic acid; PFUnDA – perfluoroundecanoic acid.


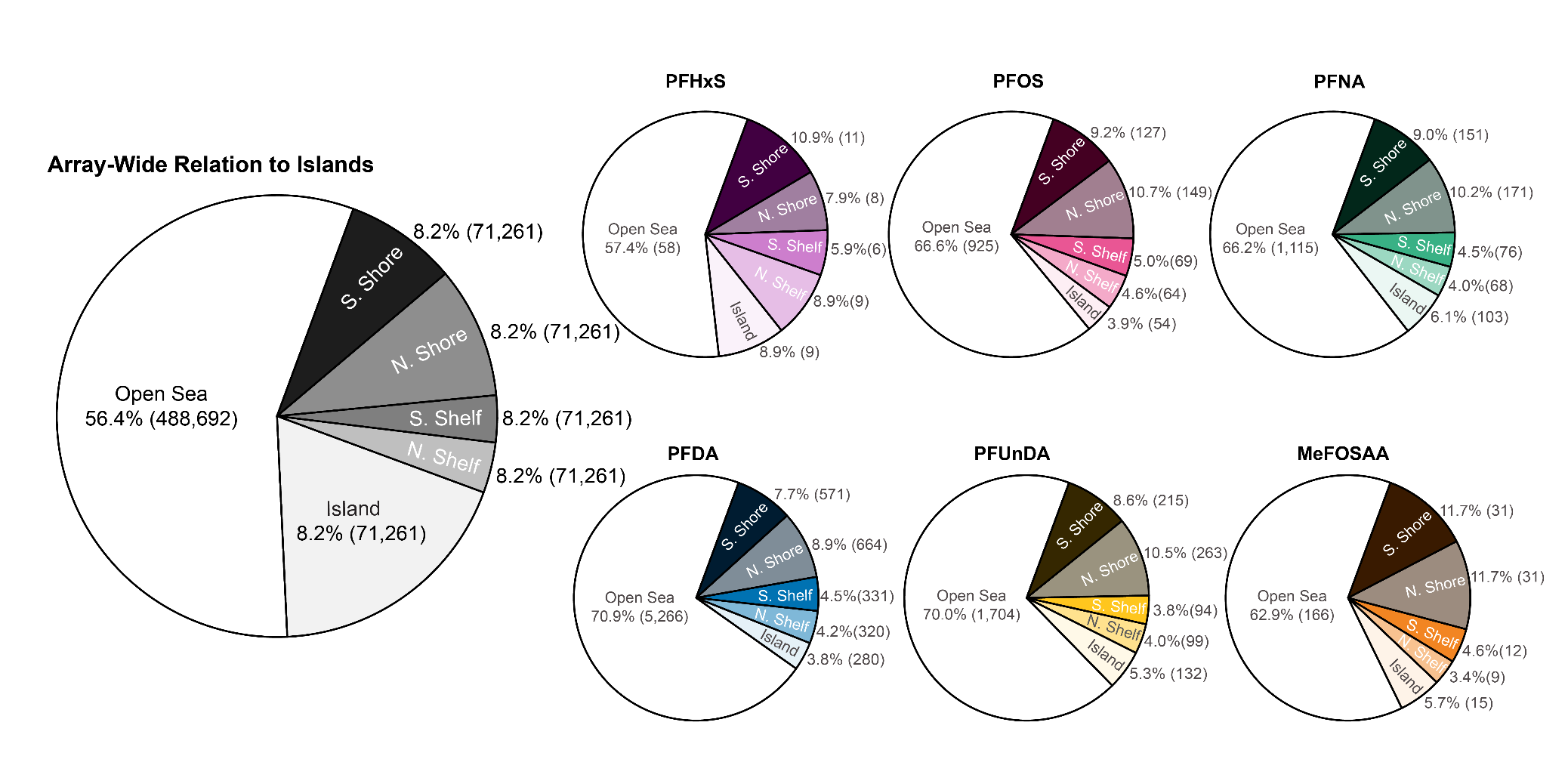
**Fig. S7: Relation to CpG islands for significant 5-hydroxymethylcytosine (5-hmC) sites by PFAS (*q*<0.05).** Each piechart shows location of significant sites in relation to CpG Islands (n=70). Charts are labeled with percent of sites in each location and the total number of sites in each category in parentheses. Open Sea; Island; N. Shelf (North Shelf); S. Shelf (South Shelf); N. Shore (North Shore); S. Shore (South Shore). Abbreviations: MeFOSAA – 2-(N-methyl-perfluorooctane sulfonamido) acetic acid; PFHxS – perfluorohexanesulphonic acid; PFDA – perfluorodecanoic acid; PFNA – perfluorononanoic acid; PFOS – perfluorooctanesulfonic acid; PFUnDA – perfluoroundecanoic acid.

**Table S7: KEGG Pathways enriched among significant 5-hmC results (*p*<0.05 and >2 differentially methylated genes, n=70)**

| **PFAS** | **KEGG ID** | **Classification** | **Description** | **Genes in Pathway** | **Significant Genes** | ***p*-value** |
| --- | --- | --- | --- | --- | --- | --- |
| PFHxS |  |  |  |  |  |  |
|  | path:hsa04110 | Cell growth and death | Cell cycle | 126 | 3 | 0.0121 |
|  | path:hsa04218 | Cell growth and death | Cellular senescence | 155 | 3 | 0.0411 |
|  | path:hsa04340 | Signal transduction | Hedgehog signaling pathway | 56 | 2 | 0.0479 |
|  | path:hsa04724 | Nervous system | Glutamatergic synapse | 114 | 3 | 0.0374 |
|  | path:hsa05218 | Cancer: specific types | Melanoma | 72 | 3 | 0.0056 |
|  | path:hsa05219 | Cancer: specific types | Bladder cancer | 41 | 2 | 0.0200 |
|  | path:hsa05224 | Cancer: specific types | Breast cancer | 147 | 3 | 0.0363 |
|  | path:hsa05226 | Cancer: specific types | Gastric cancer | 149 | 3 | 0.0399 |
| PFOS |  |  |  |  |  |  |
|  | path:hsa00562 | Carbohydrate metabolism | Inositol phosphate metabolism | 73 | 9 | 0.0419 |
|  | path:hsa04724 | Nervous system | Glutamatergic synapse | 114 | 14 | 0.0398 |
|  | path:hsa05014 | Neurodegenerative disease | Amyotrophic lateral sclerosis | 351 | 24 | 0.0371 |
|  | path:hsa05016 | Neurodegenerative disease | Huntington disease | 292 | 24 | 0.0089 |
| PFNA |  |  |  |  |  |  |
|  | path:hsa04530 | Cellular community - eukaryotes | Tight junction | 169 | 19 | 0.0367 |
|  | path:hsa04140 | Transport and catabolism | Autophagy - animal | 140 | 18 | 0.0160 |
|  | path:hsa04024 | Signal transduction | cAMP signaling pathway | 221 | 30 | 0.0013 |
|  | path:hsa04022 | Signal transduction | cGMP-PKG signaling pathway | 166 | 23 | 0.0056 |
|  | path:hsa04150 | Signal transduction | mTOR signaling pathway | 155 | 21 | 0.0059 |
|  | path:hsa04371 | Signal transduction | Apelin signaling pathway | 139 | 19 | 0.0099 |
|  | path:hsa05414 | Cardiovascular disease | Dilated cardiomyopathy | 95 | 14 | 0.0284 |
|  | path:hsa04934 | Endocrine and metabolic disease | Cushing syndrome | 155 | 20 | 0.0258 |
|  | path:hsa05146 | Infectious disease: parasitic | Amoebiasis | 100 | 16 | 0.0023 |
|  | path:hsa05145 | Infectious disease: parasitic | Toxoplasmosis | 110 | 14 | 0.0357 |
|  | path:hsa05166 | Infectious disease: viral | Human T-cell leukemia virus 1 infection | 219 | 25 | 0.0214 |
|  | path:hsa05010 | Neurodegenerative disease | Alzheimer disease | 370 | 33 | 0.0249 |
|  | path:hsa05016 | Neurodegenerative disease | Huntington disease | 292 | 26 | 0.0273 |
|  | path:hsa05022 | Neurodegenerative disease | Pathways of neurodegeneration - multiple diseases | 462 | 41 | 0.0224 |
|  | path:hsa00500 | Carbohydrate metabolism | Starch and sucrose metabolism | 35 | 5 | 0.0454 |
|  | path:hsa00470 | Metabolism of other amino acids | D-Amino acid metabolism | 6 | 2 | 0.0457 |
|  | path:hsa04211 | Aging | Longevity regulating pathway | 89 | 13 | 0.0317 |
|  | path:hsa04261 | Circulatory system | Adrenergic signaling in cardiomyocytes | 148 | 19 | 0.0356 |
|  | path:hsa04270 | Circulatory system | Vascular smooth muscle contraction | 134 | 16 | 0.0414 |
|  | path:hsa04970 | Digestive system | Salivary secretion | 92 | 13 | 0.0103 |
|  | path:hsa04971 | Digestive system | Gastric acid secretion | 76 | 12 | 0.0261 |
|  | path:hsa04972 | Digestive system | Pancreatic secretion | 101 | 12 | 0.0373 |
|  | path:hsa04911 | Endocrine system | Insulin secretion | 86 | 17 | 0.0008 |
|  | path:hsa04915 | Endocrine system | Estrogen signaling pathway | 137 | 20 | 0.0027 |
|  | path:hsa04916 | Endocrine system | Melanogenesis | 101 | 17 | 0.0023 |
|  | path:hsa04918 | Endocrine system | Thyroid hormone synthesis | 75 | 14 | 0.0016 |
|  | path:hsa04925 | Endocrine system | Aldosterone synthesis and secretion | 98 | 19 | 0.0007 |
|  | path:hsa04928 | Endocrine system | Parathyroid hormone synthesis, secretion and action | 106 | 19 | 0.0020 |
|  | path:hsa04935 | Endocrine system | Growth hormone synthesis, secretion and action | 119 | 20 | 0.0025 |
|  | path:hsa04927 | Endocrine system | Cortisol synthesis and secretion | 65 | 13 | 0.0035 |
|  | path:hsa04929 | Endocrine system | GnRH secretion | 64 | 12 | 0.0093 |
|  | path:hsa04922 | Endocrine system | Glucagon signaling pathway | 105 | 14 | 0.0149 |
|  | path:hsa04926 | Endocrine system | Relaxin signaling pathway | 129 | 17 | 0.0223 |
|  | path:hsa04919 | Endocrine system | Thyroid hormone signaling pathway | 121 | 17 | 0.0298 |
|  | path:hsa04062 | Immune system | Chemokine signaling pathway | 192 | 22 | 0.0117 |
|  | path:hsa04664 | Immune system | Fc epsilon RI signaling pathway | 67 | 10 | 0.0349 |
|  | path:hsa04672 | Immune system | Intestinal immune network for IgA production | 45 | 6 | 0.0331 |
|  | path:hsa04720 | Nervous system | Long-term potentiation | 67 | 11 | 0.0266 |
|  | path:hsa04728 | Nervous system | Dopaminergic synapse | 131 | 18 | 0.0239 |
|  | path:hsa04725 | Nervous system | Cholinergic synapse | 113 | 16 | 0.0420 |
| PFDA |  |  |  |  |  |  |
|  | path:hsa04810 | Cell motility | Regulation of actin cytoskeleton | 217 | 69 | 0.0307 |
|  | path:hsa04152 | Signal transduction | AMPK signaling pathway | 119 | 41 | 0.0270 |
|  | path:hsa04350 | Signal transduction | TGF-beta signaling pathway | 94 | 31 | 0.0175 |
|  | path:hsa04668 | Signal transduction | TNF signaling pathway | 112 | 35 | 0.0168 |
|  | path:hsa00970 | Translation | Aminoacyl-tRNA biosynthesis | 43 | 17 | 0.0075 |
|  | path:hsa04933 | Endocrine and metabolic disease | AGE-RAGE signaling pathway in diabetic complications | 100 | 34 | 0.0246 |
|  | path:hsa05330 | Immune disease | Allograft rejection | 35 | 13 | 0.0263 |
|  | path:hsa05131 | Infectious disease: bacterial | Shigellosis | 244 | 73 | 0.0064 |
|  | path:hsa05132 | Infectious disease: bacterial | Salmonella infection | 248 | 70 | 0.0141 |
|  | path:hsa05140 | Infectious disease: parasitic | Leishmaniasis | 74 | 24 | 0.0111 |
|  | path:hsa05170 | Infectious disease: viral | Human immunodeficiency virus 1 infection | 211 | 62 | 0.0114 |
|  | path:hsa00770 | Metabolism of cofactors and vitamins | Pantothenate and CoA biosynthesis | 21 | 9 | 0.0340 |
|  | path:hsa04911 | Endocrine system | Insulin secretion | 86 | 34 | 0.0083 |
|  | path:hsa04929 | Endocrine system | GnRH secretion | 64 | 25 | 0.0351 |
|  | path:hsa04960 | Excretory system | Aldosterone-regulated sodium reabsorption | 37 | 16 | 0.0193 |
|  | path:hsa04666 | Immune system | Fc gamma R-mediated phagocytosis | 96 | 36 | 0.0100 |
|  | path:hsa04664 | Immune system | Fc epsilon RI signaling pathway | 67 | 25 | 0.0214 |
|  | path:hsa04670 | Immune system | Leukocyte transendothelial migration | 113 | 35 | 0.0399 |
| PFUnDA |  |  |  |  |  |  |
|  | path:hsa00532 | Glycan biosynthesis and metabolism | Glycosaminoglycan biosynthesis - chondroitin sulfate / dermatan sulfate | 20 | 6 | 0.0176 |
|  | path:hsa05418 | Cardiovascular disease | Fluid shear stress and atherosclerosis | 139 | 19 | 0.0413 |
| MeFOSAA |  |  |  |  |  |  |
|  | path:hsa04115 | Cell growth and death | p53 signaling pathway | 73 | 3 | 0.0490 |
